# Development of an itaconic acid production process with Ustilaginaceae on alternative feedstocks

**DOI:** 10.1186/s12896-023-00802-9

**Published:** 2023-09-03

**Authors:** Paul-Joachim Niehoff, Waldemar Müller, Johannes Pastoors, Katharina Miebach, Philipp Ernst, Johannes Hemmerich, Stephan Noack, Nick Wierckx, Jochen Büchs

**Affiliations:** 1 AVT - Biochemical Engineering, RWTH Aachen University, Forckenbeckstraße 51, 52074 Aachen, Germany; 2 Institute of Bio- and Geosciences IBG-1: Biotechnology, Forschungszentrum Jülich GmbH, Wilhelm-Johnen-Straße, 52428 Jülich, Germany

**Keywords:** Ustilaginaceae, *Ustilago cynodontis*, *Ustilago maydis*, Itaconic acid, Molasses, Thick juice, Alternative feedstock, Complex media, Oxygen transfer rate

## Abstract

**Background:**

Currently, *Aspergillus terreus* is used for the industrial production of itaconic acid. Although, alternative feedstock use in fermentations is crucial for cost-efficient and sustainable itaconic acid production, their utilisation with *A. terreus* most often requires expensive pretreatment. Ustilaginacea are robust alternatives for itaconic acid production, evading the challenges, including the pretreatment of crude feedstocks regarding reduction of manganese concentration, that *A. terreus* poses.

**Results:**

In this study, five different *Ustilago* strains were screened for their growth and production of itaconic acid on defined media. The most promising strains were then used to find a suitable alternative feedstock, based on the local food industry. *U. cynodontis* ITA Max pH, a highly engineered production strain, was selected to determine the biologically available nitrogen concentration in thick juice and molasses. Based on these findings, thick juice was chosen as feedstock to ensure the necessary nitrogen limitation for itaconic acid production. *U. cynodontis* ITA Max pH was further characterised regarding osmotolerance and product inhibition and a successful scale-up to a 2 L stirred tank reactor was accomplished. A titer of 106.4 g_itaconic acid_/L with a theoretical yield of 0.50 g_itaconic acid_/g_sucrose_ and a space-time yield of 0.72 g_itaconic acid_/L/h was reached.

**Conclusions:**

This study demonstrates the utilisation of alternative feedstocks to produce ITA with Ustilaginaceae, without drawbacks in either titer or yield, compared to glucose fermentations.

**Supplementary Information:**

The online version contains supplementary material available at 10.1186/s12896-023-00802-9.

## Background

Due to the increased demand for bulk chemicals, bio-based production of these molecules plays a significant role in the transition towards a circular bioeconomy [1]. Carboxylic acids, amines, and alcohols are already being produced via fermentation. However, their market share was only 1–2% in 2019 [2]. One reason are the high production costs of bio-based chemicals, to which the feedstock is an essential factor [3, 4]. Alternative feedstocks from renewable sources can decrease production costs and increase sustainability. Especially waste and side streams from local industries show high potential for circular biorefinery concepts [1, 5, 6].

Different carboxylic acids, like citric, succinic, or lactic acid, were already produced using alternative feedstocks [7–11]. Itaconic acid (ITA) is another carboxylic acid of interest, due to its two functional groups and an expected market size of US$ 117.1 M [12]. Esterification of the carboxyl groups and polymerisation of the methyl group allows for a broad range of application, for example as hydrogels, superabsorbers or as additives in industrial adhesives [13–18]. In addition, 3-methyl tetrahydrofuran – a potential biofuel – can be produced from ITA [19]. Interestingly, ITA has also been identified within the human immune response, leading to applications as therapeutic agent [20, 21].

Industrial production of ITA is, until now, exclusively realised using *Aspergillus terreus*, due to its tolerance to low pH and high titers and yields [22–25]. Industrially relevant titers of up to 160 g/L in pulsed batch fermentations and yields of up to 0.58 g_ITA_/g_glucose_ are reported in literature [22]. However, there are several disadvantages using this strain. Its filamentous morphology poses challenges with oxygenation, shear stress during fermentation, and risk of failed batches [26]. In addition, the pretreatment of complex substrates is necessary for high productivity in this strain. For example, even trace amounts of manganese are highly detrimental to ITA formation [25, 27]. Finally, the pathogenic classification of *A. terreus* limits its applicability even further [28, 29].

To evade these challenges, alternative organisms for ITA production have become the focus of research. These include genetically engineered organisms like *Corynebacterium glutamicum* [30] or *Escherichia coli* [31] and natural ITA producers like *Ustilago sp.* [32] or *Candida sp*. [33]. Of the latter, especially *Ustilago sp*. have found increasing recognition in recent years [32, 34–36]. Two representatives, *U. maydis* and the pH tolerant *U. cynodontis*, show great potential for tackling the challenges presented for ITA production with *A. terreus*. As plant pathogens infecting corn plants, they do not affect animal health [29]. Furthermore, through genetic engineering, yeast-like morphology can be sustained throughout the entire fermentation, allowing for better oxygenation and lower susceptibility to hydromechanical stress than *A. terreus* [36, 37]. Moreover, unwanted side-product formation was deleted [38]. By introducing additional feed pulses, titers of up to 220 g/L (pulsed batch fermentation with *in -situ* crystallisation) and 83 g/L (pulsed-batch fermentation) with yields of up to 0.61 g_ITA_/g_glucose_ and 0.39 g_ITA_/g_glucose_ were reached for *U. maydis* and *U. cynodontis*, respectively [37, 39]. Deep metabolic and morphological engineering further enabled itaconic acid production at the maximum theoretical yield in a fermentation with constant glucose feed [40]. However, these key performance indicators (KPI) were reached in defined media. While some years ago, *U. maydis* has been shown to produce ITA from alternative feedstocks, like beech wood or brewers’ spent grain, only recently such research was published for *U. cynodontis* [41–45].

Alternative feedstocks are inherently complex and contain a plethora of compounds besides the carbon source. These include, for example, additional nitrogen sources, pigments, or salts. While pigments may require additional purification steps in downstream processing, different salts have been shown to be detrimental to ITA production [22, 26, 46]. In addition, organic acids, often produced by microbial activity during storage of the feedstock, can negatively influence growth and product formation in the production process [47]. Because both *Ustilago* strains used in this study need a nitrogen limitation to initiate ITA production [48], the biologically available nitrogen content of the alternative feedstock is of utmost importance and needs to be evaluated. In addition, the use of complex substrates increases the osmolality of the medium, which might lead to prolonged lag phases or even prevent growth [49]. Therefore, the choice of substrate is crucial for the feasibility of the fermentation process.

The oxygen transfer rate (OTR) is a crucial parameter for microbial cultivations. It allows the determination of biological phenomena like diauxic growth, substrate limitations or oxygen limitation, as it is directly coupled to the metabolic activity of the microbe [50]. It has repeatedly been used for the investigation of microbial growth and product formation and is, therefore, the perfect parameter for bioprocess development [51–53].

In this study, five strains of *U. maydis* and *U. cynodontis* were screened regarding growth and product formation on defined media. One *U. cynodontis* and one *U. maydis* strain were then further used to investigate different side and waste streams from local food industry, to find a suitable substrate for ITA production. In the next step, the carbon preference of the production organism was analysed and the biologically available nitrogen content of thick juice and molasses was determined. With knowledge of the influence of osmolality and pH on ITA production, a scale-up from shake flasks to a 2 L stirred tank reactor (STR) was realised to conclude this study.

## Results

### Screening for a suitable production organism and feedstock

**Fig. 1:**
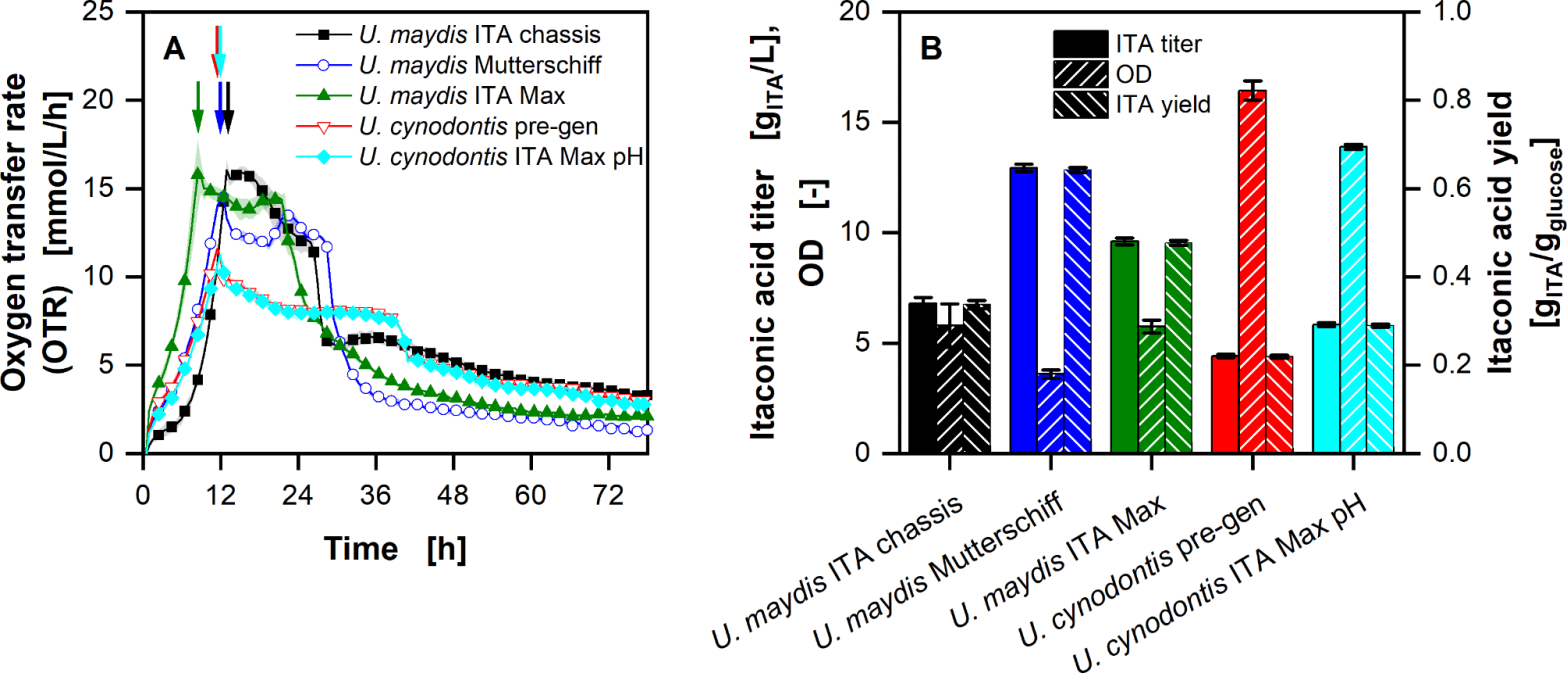
**Growth and production of different Ustilaginaceae strains grown on glucose with limiting ammonium chloride concentration (1 g/L NH4Cl)**. **(A)** Course of the oxygen transfer rates. Arrows indicate start of nitrogen limitation. **(B)** Itaconic acid titers, optical densities and yields of ITA on glucose after 78 h of cultivation. Cultivations were performed in a 96 round deep-well MTP, filled with 300 µL modified Verduyn medium with 25 g/L glucose at 30 °C, 350 rpm shaking frequency and a shaking diameter of 50 mm. 100 mM and 30 mM MES were added to the *U. maydis* and *U. cynodontis* cultivations, respectively. The initial pH was set to 6.5 for all cultures. Graphs in (A) show the mean of three replicates, with standard deviation as shaded area. Due to high reproducibility of the measurements, the standard deviation might not be visible for every data point. For clarity, only every fourth data point is shown

To compare the five available strains (two *U. cynodontis* and three *U. maydis* strains) under the same conditions in a time efficient manner, the newly developed µTOM for online oxygen transfer rate (OTR) measurment was used for an experiment with glucose as carbon source (Fig. 1) [54]. The µTOM allows the parallel measurement in a 96 well microtiter plate. To enable ITA production, the cultivation was performed under nitrogen-limiting conditions.

All five strains show three distinct phases: an exponential growth phase, a nitrogen-limited production phase, and a carbon depletion phase. The typical phases of ITA production with Ustilaginaceae on glucose are illustrated in Figure S1. During the first phase, the OTR increases exponentially, until a drop is visible. During the following phase, the Ustilaginaceae strains behave differently. The OTR of *U. maydis* ITA chassis shows a short plateau at 15.8 mmol/L/h, before it decreases to 6.5 mmol/L/h after 26 h. Both other *U. maydis* strains show an OTR that first decreases and then increases again after the exponential growth phase. The OTR of both *U. cynodontis* strains decreases slowly and then remains constant at ~ 8 mmol/L/h. Once the glucose is depleted, the OTR drops sharply for all strains. The total production time for *U. maydis* Mutterschiff is 14 h, while *U. cynodontis* ITA Max needed 28 h to consume all substrate. At the end of the cultivation, *U. maydis* ITA chassis and *U. maydis* ITA Max reached an optical density (OD) of 5.8 (no significant difference) and ITA titers of 6.8 and 9.8 g/L, respectively. *U. maydis* Mutterschiff showed the lowest OD of 3.6 and the highest titer of 12.9 g/L. At the same time, *U. cynodontis* pre-gen and *U. cynodontis* ITA Max pH reached an OD of 16.4 and 13.9 and ITA titers of 4.4 and 5.9 /L, respectively.

**Fig. 2:**
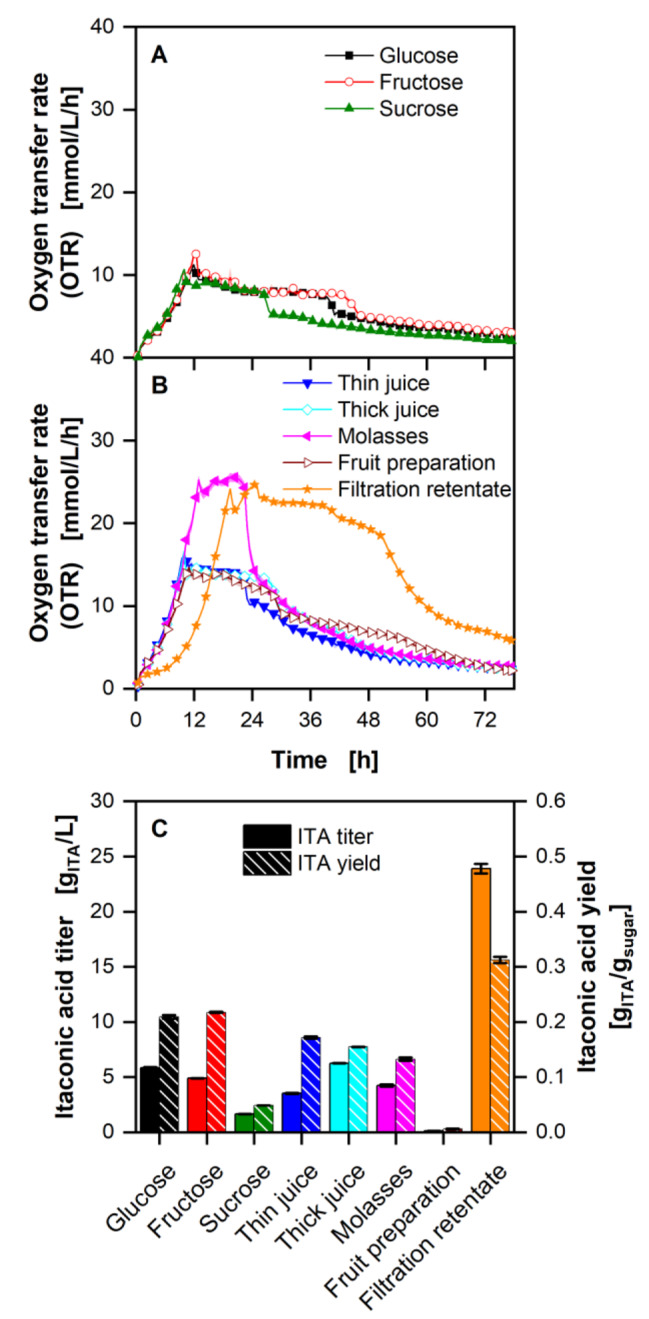
**Cultivation of *U. cynodontis * ITA Max pH with different carbon sources with limiting ammonium chloride concentration (1 g/L NH4Cl)**. **(A)** Course of the oxygen transfer rates of the pure sugars (glucose, fructose and sucrose). **(B)** Oxygen transfer rates of complex substrates. **(C)** Itaconic acid titers and yields of the cultivations after 78 h of cultivation. Cultivations were performed in a 96 round deep-well MTP, filled with 300 µL modified Verduyn medium at 30 °C, 350 rpm shaking frequency and a shaking diameter of 50 mm. 30 mM MES were added to the cultivations. The initial pH was set to 6.5 for all cultivations. The different carbon sources are specified in supplementary Table S1. Graphs show the mean of three replicates, with standard deviation as shaded area. Due to high reproducibility of the measurements, the standard deviation might not be visible for every data point. For clarity, only every fourth data point is shown

Figure 2 shows the OTR, ITA titers and yields of *U. cynodontis* ITA Max pH on different carbon sources (compare Table S1). On the pure sugars glucose, fructose and sucrose, the highest OTR reached is 12.5 mmol/L/h, which is considerably lower than the OTR reached with complex carbon sources. OTRs of ~ 13.5 mmol/L/h were reached with sugar beet thin juice, thick juice, and fruit preparation and ~ 25.0 mmol/L/h with molasses and the filtration retentate.

ITA production is shown in Fig. 2C with all carbon sources, except filtration permeate (data not shown). While titers of 23.9 g/L were reached with the filtration retentate, lower titers of 0.1 g/L could be reached with the fruit preparation. Even higher titers were reached with *U. maydis* Mutterschiff, while the strain was also able to produce increased amounts of ITA from the fruit preparation (Figure S2). For *U. cynodontis* ITA Max pH the highest yield of 0.30 g_ITA_/g_sugar_ was reached with filtration retentate, even outperforming production on glucose and fructose with yields of 0.22 and 0.21 g_ITA_/g_sugar_ (no significant difference), respectively. The yields from thin- and thick juice and molasses are between 0.13 and 0.17 g_ITA_/g_sugar_, with thin juice showing the highest and molasses the lowest yield.

**Fig. 3:**
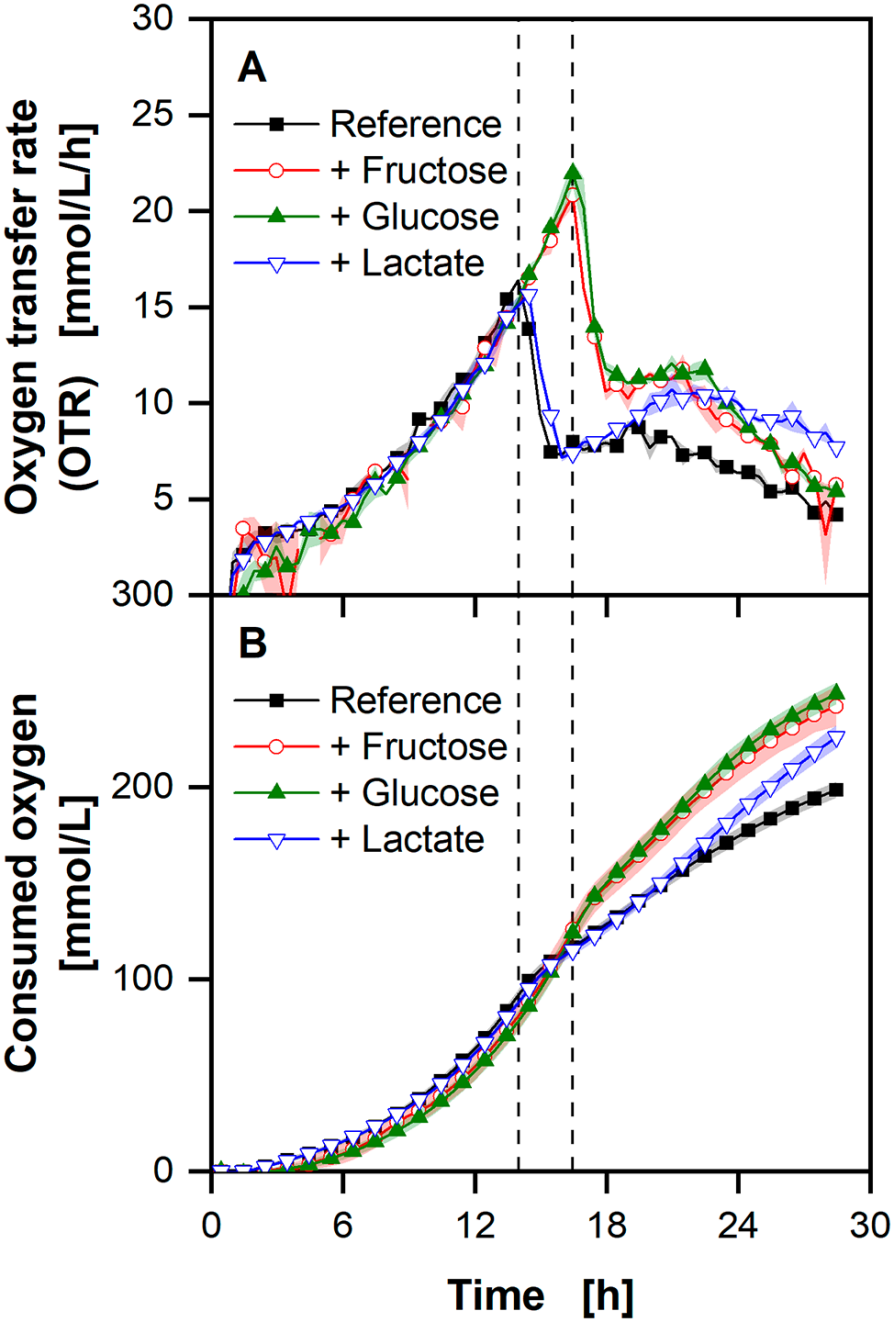
**Growth of *U. cynodontis*** **ITA Max pH on different carbon sources available in complex substrates without nitrogen limitation**. The basic medium contained 5 g/L fructose, glucose and lactate each, denoted as “Reference”. The concentration of fructose, glucose and lactate was each increased to 10 g/L. The curves in the figure are denoted by the type of additional carbon source. **(A)** Course of the oxygen transfer rates. **(B)** Consumed oxygen (integral of the data in (A)). Dashed vertical lines show the depletion of the fructose and glucose. The first dashed line shows the depletion of fructose and glucose for the reference and the cultivation with increased lactate, while the second dashed line shows the depletion for the cultivations with increased sugars. Cultivations were performed in 250 mL RAMOS flasks filled with 10 mL modified Verduyn medium at 30 °C, 350 rpm shaking frequency and a shaking diameter of 50 mm. 30 mM MES were added to the cultivations. The initial pH was set to 6.5 for all cultivations. The final pH reached 6.37 ± 0.02, 6.22 ± 0.01, 6.23 ± 0.02, and 7.56 ± 0.03 for the reference, increased fructose, glucose and lactate cultivations, respectively. Errors describe the deviation of the maximum and minimum pH value from the mean of duplicates. Graphs show the mean of duplicates. Shadows show the minimum and maximum values. Due to high reproducibility of the measurements, the standard deviation might not be visible for every data point. For clarity, only every second data point is shown

To further analyse the possible substrates, *U. cynodontis* ITA Max pH was grown on different monomeric carbon sources available in thick juice and molasses (Fig. 3).

The OTR of all four cultivations show similar behaviour with an exponential increase, until a sharp drop, which comes after 14 h for the reference and the cultivation with additional lactate. It is delayed by 2 h for the experiments with additional sugars (glucose and fructose). After the drop, the OTR increases slightly for the reference and the cultivations with additional sugars before slowly decreasing until the cultivations’ end. Additional lactate prolongs the increase after the first OTR drop, compared to the reference. Correlating to the decline in the OTR (Fig. 3A), the increase in the consumed oxygen decreases (Fig. 3B). While it is similar for both runs with increased monosaccharide concentration, consumed oxygen for the reference and the cultivation with additional lactate differs after the drop. At the end of the cultivation 250 mmol/L oxygen were consumed in the cultivations with added monosaccharides, 226 mmol/L with increased lactate, and 198 mmol/L in the reference. The pH at the end of the cultivations with additional sugars is lower than the reference, which in turn is significantly lower than the one with increased lactate.

### Determination of biologically available nitrogen in molasses and thick juice

Due to the necessary nitrogen limitation, nitrogen availability in the feedstock plays a major role [48]. Therefore, the available nitrogen content in the complex substrate needs to be determined. The maximum OTR (OTR_N,max_) is reached, when the nitrogen limitation starts. A correlation between nitrogen concentration in the medium and the OTR_N,max_ can be obtained on defined minimal media, with known nitrogen concentrations. This correlation can then be used, to determine the unknown biologically available nitrogen concentration in thick juice and molasses.

**Fig. 4:**
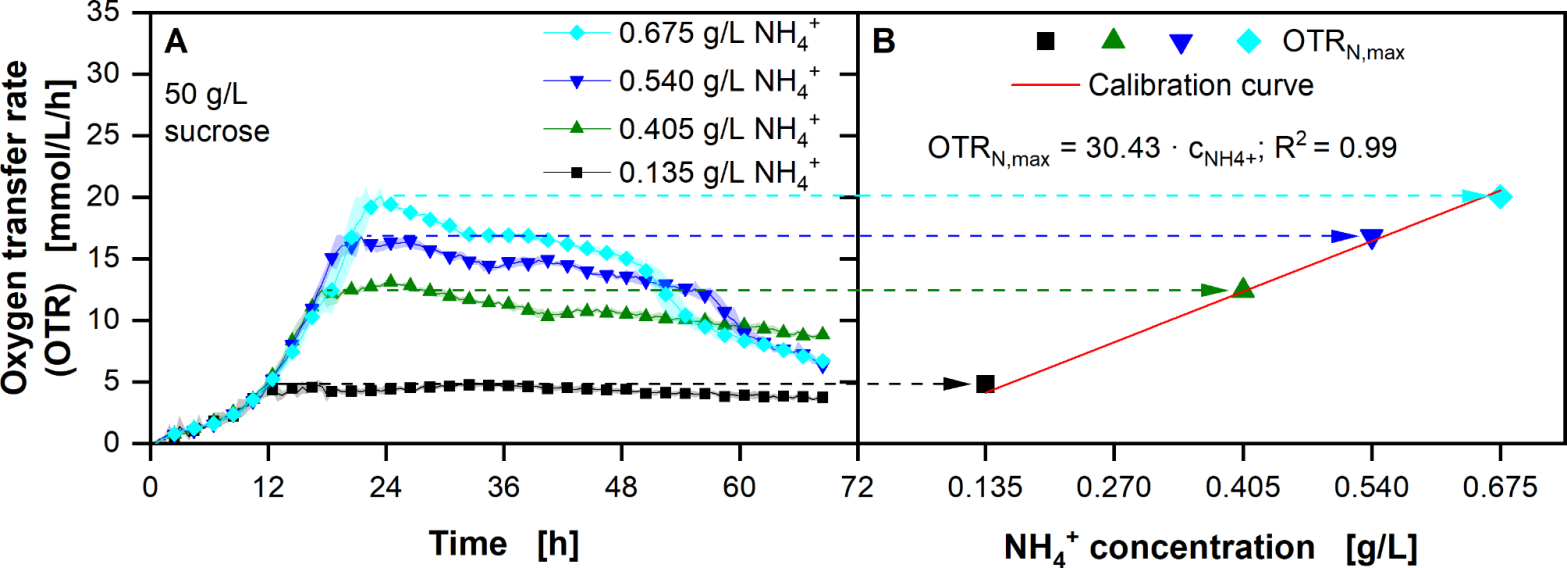
**Determination of a correlation between the maximum obtained oxygen transfer rate (OTR**_**N,max**_**) and ammonium ion (NH**_**4**_^**+**^**) concentration using** ***U. cynodontis*** **ITA Max pH**. **(A)** Course of the oxygen transfer rates with addition of specific NH_4_^+^ concentrations in modified Verduyn medium. **(B)** Correlation between OTR_N,max_ and NH_4_^+^ concentration. Cultivations were performed in a 96 round deep-well MTP, filled with 300 µL modified Verduyn medium with 50 g/L sucrose and 0.135-0.675 g/L NH_4_^+^ (0.4-﻿2.0 g/L NH_4_Cl) at 30 °C, 350 rpm shaking frequency and a shaking diameter of 50 mm. 30 mM MES were added to the cultivations. The initial pH was set to 6.5 for all cultivations. Graphs show the mean of three replicates, with standard deviation as shaded area. Due to high reproducibility of the measurements, the standard deviation might not be visible for every data point. For clarity, only every fourth data point is shown

*U. cynodontis* ITA Max pH was cultivated in defined medium with sucrose as carbon source (Fig. 4A). With increasing ammonium concentrations, the resulting OTR_N,max_ increases accordingly (Fig. 4B). The OTR_N,max_ reaches 4.7 mmol/L/h with 0.135 g/L ammonium and increases up to 19.8 mmol/L/h with 0.675 g/L ammonium (Fig. 4A). Fitting a linear model through the OTR_N,max_ values over the initially added ammonium ion concentration leads to a calibration curve, which can be used to determine the biologically available nitrogen concentration in any complex medium.

**Fig. 5:**
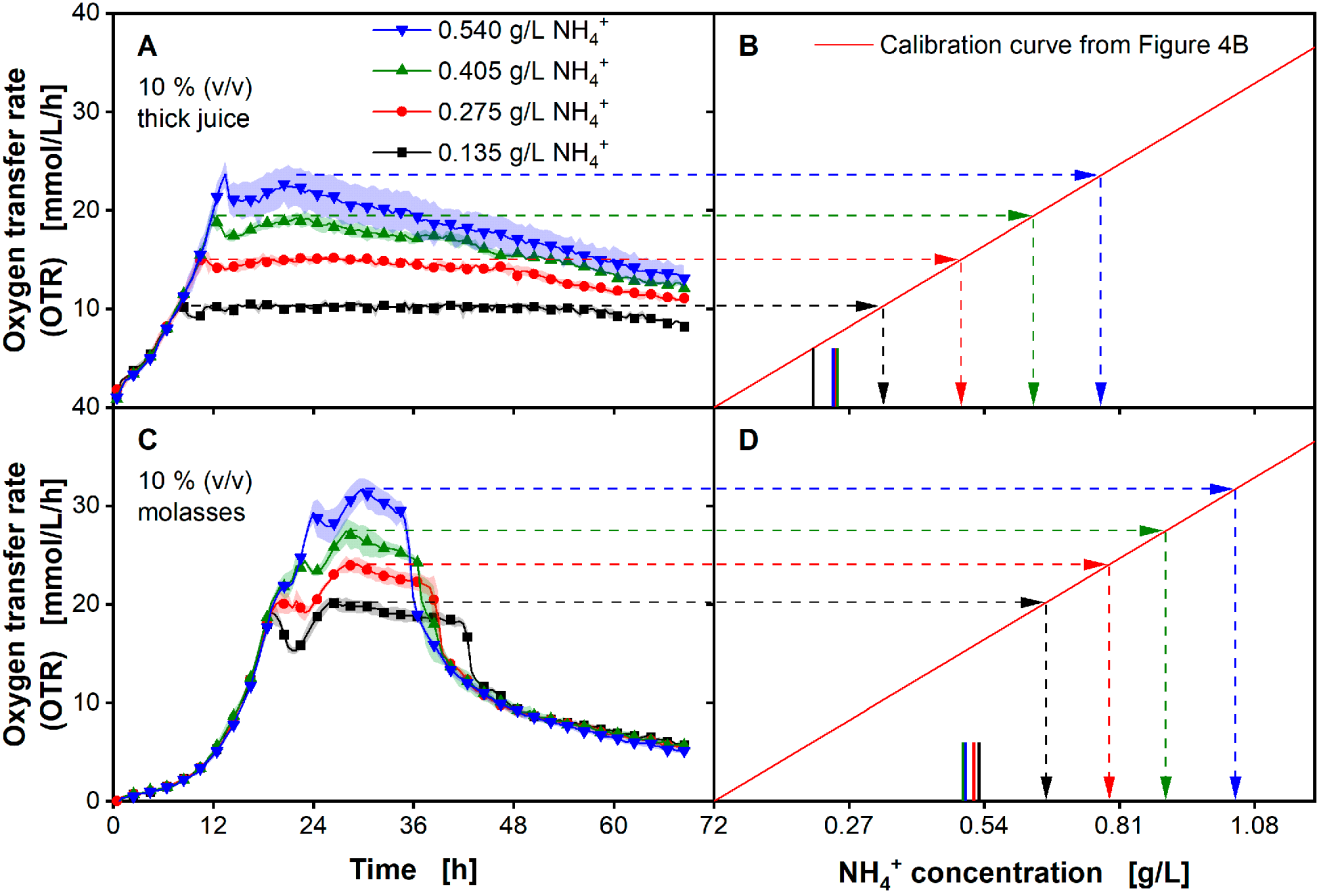
**Estimation of biologically available ammonium ion concentration equivalents in thick juice (A) and molasses (C) using** ***U. cynodontis*** **ITA Max pH**. Course of the oxygen transfer rates with addition of specific NH_4_^+^ concentrations in modified Verduyn medium. Estimated NH_4_^+^ equivalents in the medium containing thick juice **(B)** or molasses **(D)**. Arrows show available NH_4_^+^ concentration in the respective medium. Solid colored vertical lines in Fig. 5B and D indicate the biologically available NH_4_^+^ equivalents in modified Verduyn medium without added NH_4_^+^. Cultivations were performed in a 96 round deep-well MTP, filled with 300 µL modified Verduyn medium with 10% (v/v) thick juice or molasses and 0.135-0.540 g/L NH_4_^+^ (0.4-1.6 g/L NH_4_Cl) at 30 °C, 350 rpm shaking frequency and a shaking diameter of 50 mm. 30 mM MES were added to the cultivations. The initial pH was set to 6.5 for all cultivations. Graphs show the mean of three replicates, with standard deviation as shaded area. Due to high reproducibility of the measurements, the standard deviation might not be visible for every data point. For clarity, only every fourth data point is shown

In a second step, * U. cynodontis* ITA Max pH was cultivated in the same medium, while the carbon source was replaced by thick juice (Fig. 5A) or molasses (Fig. 5C). While the general course of the OTR of the thick juice cultivations looks similar to the ones on pure sucrose (Fig. 4A), the ones on molasses differ, after the initial exponential increase that lasts for 18 h (Fig. 5C). While the OTR of the lowest ammonium concentration drops, the other three stay either constant or increase. With the calibration curve from Fig. 4B, the OTR_N,max_ can be used to calculate the ammonium equivalents available in the media. From these, the initially added ammonium concentration needs to be subtracted to determine the biologically available nitrogen in the added thick juice or molasses. This leads to 2.3 ± 0.2 and 5.1 ± 0.1 g/L ammonium ion equivalents available in thick juice and molasses, as indicated by vertical lines in Fig. 5B and D, respectively.

### Influence of osmolality, pH and ITA concentrations on *U. cynodontis* cultivations

Defined media with increased osmolalities and their effect on growth and ITA production were investigated.

**Fig. 6:**
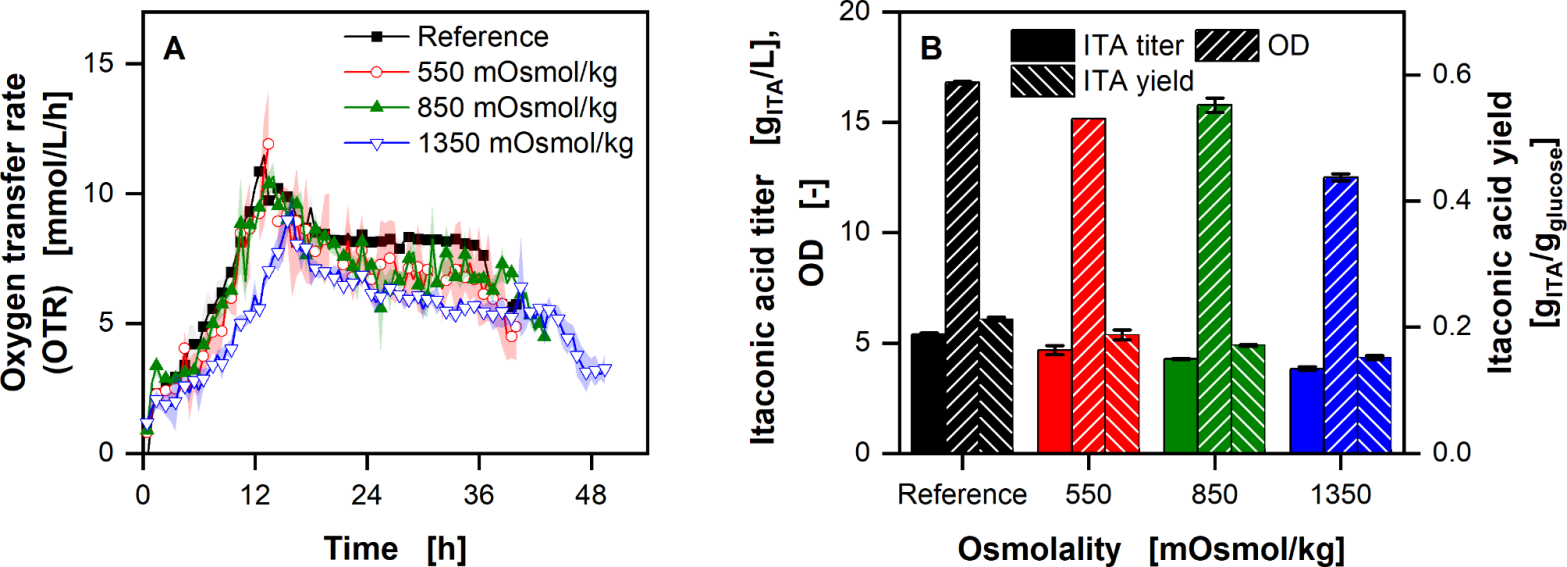
**Influence of osmolality on growth and productivity of** ***U. cynodontis*** **ITA Max pH with limiting ammonium chloride concentration (1 g/L NH4Cl)**. **(A)** Course of the oxygen transfer rates. **(B)** Itaconic acid titer, OD and yield at the end of the respective fermentations. Osmolality was increased by addition of 0.1, 0.25 and 0.5 M NaCl. The reference medium has an osmolality of 350 mOsmol/kg. Cultivations were performed in 250 mL RAMOS flasks filled with 10 mL modified Verduyn medium with 25 g/L glucose at 30 °C, 350 rpm shaking frequency and a shaking diameter of 50 mm. 30 mM MES were added to the cultivations. The initial pH was set to 6.5 for all cultivations. The final pH reached 2.80 ± 0.01, 2.77 ± 0.01, 2.70 ± 0.01, and 2.65 ± 0.01 for the reference, 550, 850 and 1350 mOsmol/kg cultivations, respectively. Graphs show the mean of duplicates. Shadows and error bars show the minimum and maximum values. For clarity, only every second data point is shown

The OTR of the reference cultivation with 350 mOsmol/kg is similar to the ones with an osmolality of up to 850 mOsmol/kg (Fig. 6A). However, higher osmolalities increase the time until the nitrogen limitation is reached by 33% for 1350 mOsmol/kg. At the same condition, the OD decreased by 26% from 16.8 ± 0.1 to 12.5 ± 0.2 (Fig. 6B). The ITA titer decreases slightly with increasing osmolality from 5.4 ± 0.1 to 3.8 ± 0.1 g/L. With decreasing product concentration, the yield also decreases from 0.21 ± 0.00 for the reference to 0.15 ± 0.00 g_ITA_/g_glucose_ with an osmolality of 1350 mOsmol/kg.

**Fig. 7:**
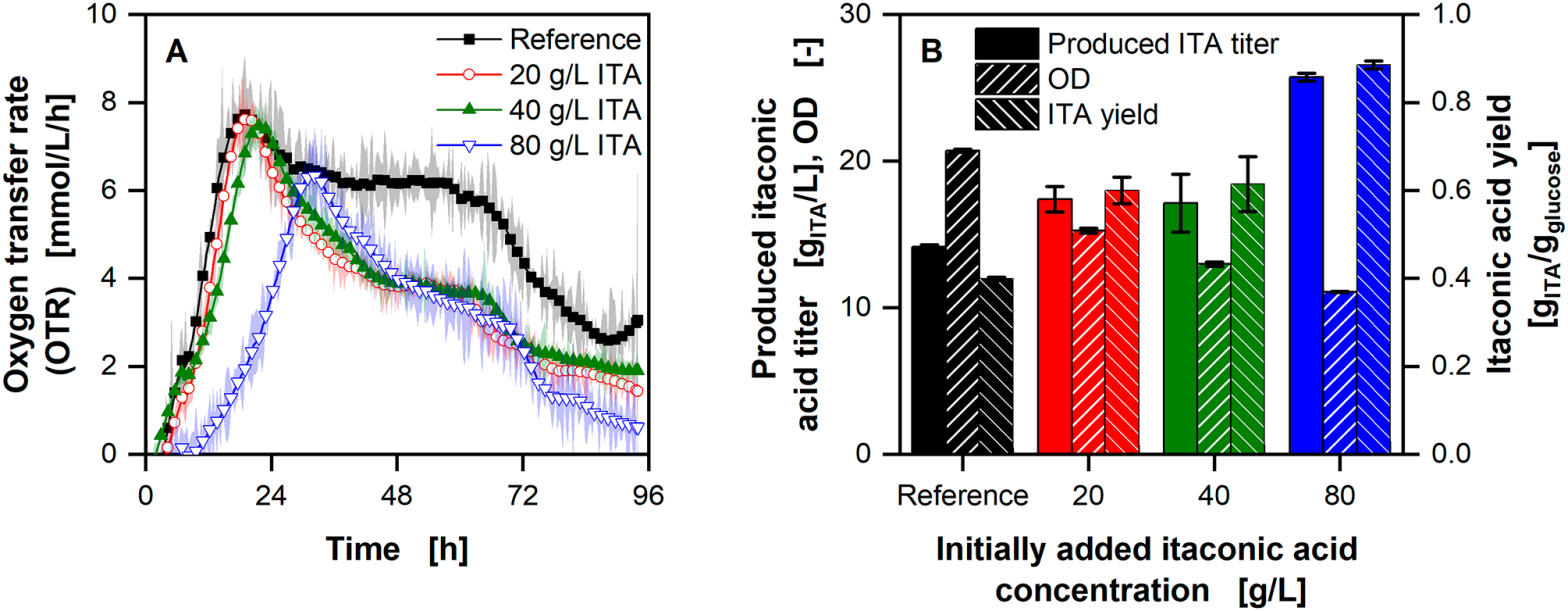
**Influence of itaconic acid concentrations on** ***U. cynodontis*** **ITA Max pH with limiting ammonium chloride concentration (1 g/L NH4Cl**). **(A)** Course of the oxygen transfer rates with increasing itaconic acid concentrations added at the beginning of the experiments. **(B)** Produced itaconic acid titer (in addition to the amount added at the beginning), OD and yield after 95 h. Cultivations were performed in 250 mL RAMOS flasks filled with 10 mL modified Verduyn medium with ~ 25 g/L glucose at 30 °C, 350 rpm shaking frequency and a shaking diameter of 50 mm. 30 mM MES were added to the cultivations. The initial pH was set to 6.5 for all cultivations. The final pH reached 1.88 ± 0.04, 4.42 ± 0.04, 5.08 ± 0.03, and 5.39 ± 0.01 for the reference, 20, 40 and 80 g/L added itaconic acid cultivations, respectively. **(A)** Graphs show the smoothed mean (Savatzky-Golay) of duplicates. Shadows show the minimum and maximum values of the unsmoothed data. **(B)** Error bars show the minimum and maximum value of duplicates. For clarity, only every fourth data point is shown

Figure 7 shows the influence of increased initial ITA concentrations on OTR, final ITA titer, OD, and yield. To differentiate between possible cytotoxic effects of the acid itself and osmolality or pH effects, the added ITA concentrations were chosen to reach the same osmolalities as seen in Fig. 6.

The OTR peaks are shifted slightly from 19 to 22 h with the addition of 40 g/L ITA, compared to the reference and addition of 20 g/L ITA. With the initial addition of 80 g/L ITA, the peak is reached after 32 h. In addition, the biomass is decreased with increased initial ITA concentrations (Fig. 7B). In the reference cultivation without the initial addition of ITA, the pH decreases to 1.88 ± 0.04. With increasing ITA concentrations, the final pH increases to 4.42 ± 0.04, 5.08 ± 0.03, and 5.39 ± 0.01 for 20, 40, and 80 g/L ITA, respectively.

### Extended batch stirred tank reactor cultivation of *U. cynodontis* ITA Max pH with thick juice as carbon source cultivations

**Fig. 8:**
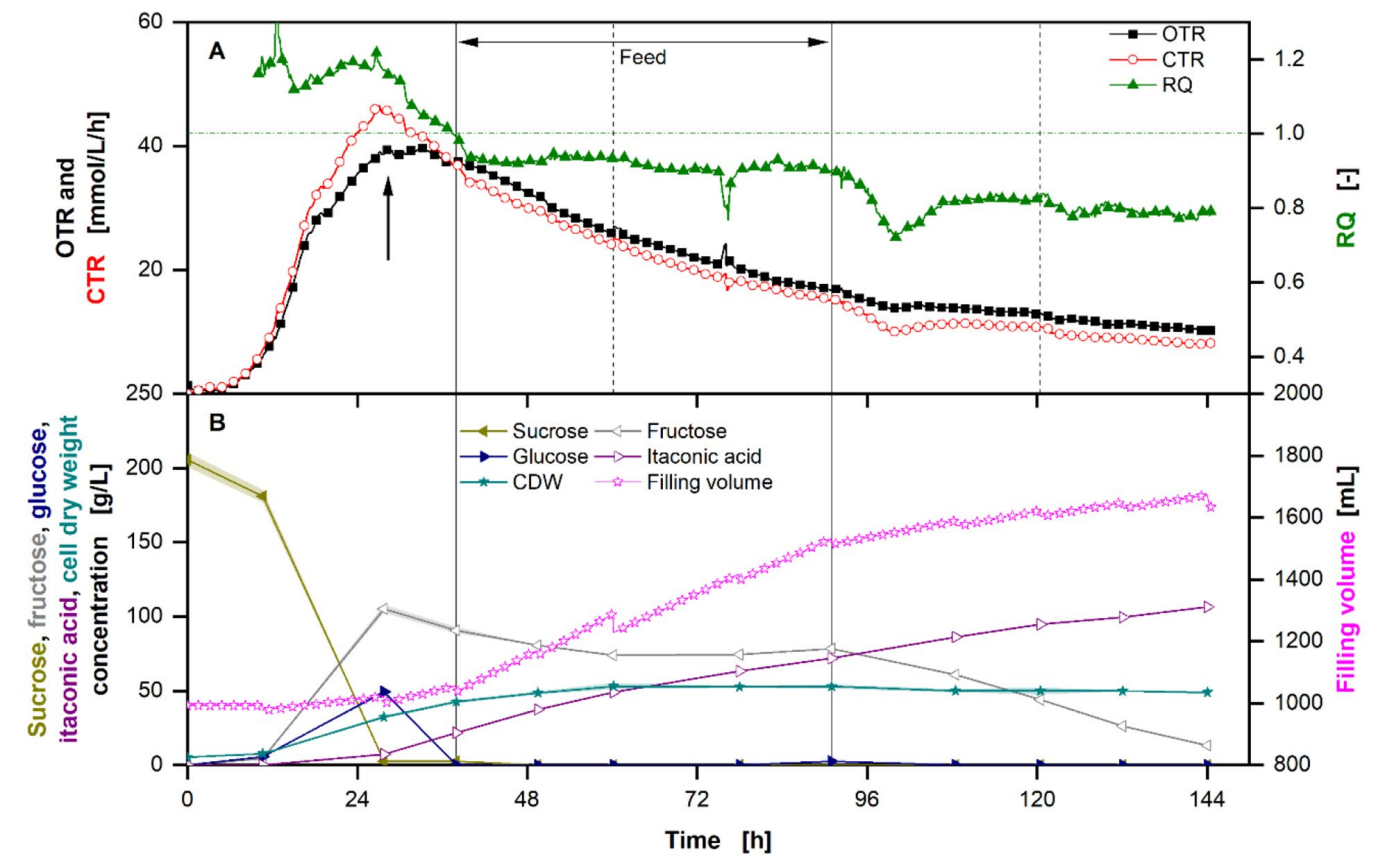
**Extended-batch fermentation of** ***U. cynodontis*** **ITA Max pH grown with thick juice as sole carbon source with limiting ammonium chloride concentration (4 g/L NH4Cl)**. **(A)** Course of the oxygen and carbon dioxide transfer rates and respiratory quotient. The black arrow indicates the start of the nitrogen limitation. The horizontal green dotted-dashed line indicates an RQ = 1. **(B)** Sugar (sucrose, glucose, fructose) and product concentrations (itaconic acid, cell dry weight) and filling volume. For the batch phase, 200 g/L sucrose from thick juice were initially added to the medium. During the feed phase (between the vertical solid lines) 200 g of additional sucrose in form of thick juice were added into the fermentation vessel. Vertical dotted lines show the addition of antifoam. In addition, antifoam was added at the end of the feed phase. Cultivation was performed in a 2 L Sartorius BIOSTAT^®^ stirred tank reactor (Sartorius AG, Göttingen, Germany) with an initial filling volume of 1 L at 30 °C. Dissolved oxygen tension was kept > 30% by increasing the stirring frequency from 800 to 1200 rpm. pH was kept constant at 6.5 by addition of 1 M HCl and 5 M NaOH. Absolute aeration rate, pH, dissolved oxygen tension and stirring frequency are shown in Figure S4. RQ values are only shown for OTR values > 5 mmol/L/h. Samples were taken regularly and analyzed via HPLC. Concentrations are shown as mean values of three replicates, and standard deviation as shaded area. Due to high reproducibility of the measurements, the standard deviation might not be visible for every data point. For clarity, only every tenth data point is shown for the online data

To demonstrate the feasibility of using thick juice as the sole carbon source on larger scale, *U. cynodontis* ITA Max pH was cultivated in a 2 L STR (Fig. 8). During the feed phase, substrate was continuously fed at non limiting carbon concentrations.

The OTR and carbon dioxide transfer rate (CTR) increase exponentially for the first 18.5 h, after which they increase linearly until 28 h (Fig. 8A). During this time, the CTR is higher than the OTR, leading to a respiratory quotient (RQ) of ~ 1.2. While the cell dry weight (CDW) increases from initially 5.4 to 32.3 g/L, sucrose is equally converted to its monomers, glucose and fructose (Fig. 8B). However, a higher accumulation of fructose can be observed after 10 h.

After 28 h, the OTR and CTR start to decrease, resulting in an RQ which decreases from 1.2 to 0.9 between 28 h and the beginning of the feed phase (Fig. 8A). At this time, the ITA concentration starts to increase, while the CDW continues to increase.

During the feed phase, thick juice and, thus, nitrogen and carbon, is fed into the reactor with a constant feed rate of 5.5 ml/h. This corresponds to 0.013 ± 0.001 g/h NH_4_^+^ equivalents. While CDW increases during this time, the cells continue to produce ITA (Fig. 8B). The RQ stays constant at 0.9. At the same time, the sugar concentrations remain almost constant, showing the immediate conversion of sucrose and consumption of glucose (Fig. 8B). However, it is noteworthy that during the whole cultivation, and especially during the feed phase, the filling volume increases from initially 1 to 1.65 L, due to feeding and pH adjustments (Fig. 8B). This leads to a constant dilution effect of the substrate, product, and CDW concentrations. Thus, the increase in fructose and CDW mass is masked by the increasing filling volume. Once the feed ends, the RQ drops to 0.8 and fructose concentrations start to decrease and reach 13.2 g/L at the end of the fermentation. In total, the production time starting from the nitrogen limitation at 28 h, was 116.2 h.

In the end, a final concentration of 106.4 ± 0.1 g/L ITA was reached. In total, 194.9 ± 0.2 g ITA with a space-time-yield (STY) of 0.72 ± 0.00 g_ITA_/L/h were produced. This corresponds to a yield of 0.48 ± 0.02 g_ITA_/g_sucrose_ or 0.46 ± 0.02 g_ITA_/g_glucose_. In a similar experiment, *U. maydis* Mutterschiff reached titers of 99.8 ± 0.7 g/L with a yield of 0.66 ± 0.02 g_ITA_/g_sucrose_ (0.63 ± 0.02 g_ITA_/g_glucose_) and a STY of 0.92 ± 0.00 g_ITA_/L/h (Figure S5). Here, a final CDW of 53 g was reached, while the production phase lasted for 125 h. In an optimised process, due to high costs, no sugar should be left in the medium. Thus, excluding the excess fructose, theoretical yields of 0.50 ± 0.02 g_ITA_/g_sucrose_ (0.48 ± 0.02 g_ITA_/g_glucose_) and 0.72 ± 0.02 g_ITA_/g_sucrose_ (0.66 ± 0.02 g_ITA_/g_glucose_) were reached with *U. cynodontis* ITA Max pH and *U. maydis* Mutterschiff, respectively.

## Discussion

### Screening for a suitable production organism and feedstock

Production of ITA is generally possible with both *U. maydis* and *U. cynodontis*, and each of these has their own unique features that are relevant for process development [55]. Therefore, finding a suitable production strain is the first step in establishing a production process for ITA on alternative feedstocks. However, using complex substrates for cultivations introduces a new set of challenges, not seen with defined media [56]. These include impurities or excessive amounts of nitrogen sources, which might negatively influence the productivity of the organism. Therefore, first strain selection was performed on defined medium.

All tested strains show exponential growth, until a drop indicates the onset of the nitrogen limitation, as described before (Fig. 1) [26]. Once the nitrogen limitation starts, ITA production is induced [48]. During the production phase *U. maydis* ITA Max and *U. maydis* Mutterschiff show an OTR that first decreases and then increases again, hinting at a change in metabolism during the production phase. Nevertheless, *U. maydis* Mutterschiff reaches the highest ITA titers, compared to the other two *U. maydis* strains. These results are not surprising, given the fact that *U. maydis* Mutterschiff is the most genetically engineered of the three strains. All three *U. maydis* strains have the *fuz7* and *cyp3* genes deleted, to allow for yeast like growth and hinder the transformation of ITA to 2-hydroxyparaconate. In addition, the strains have an overexpression of the *ria* gene, which regulates the whole ITA gene cluster [37, 38, 40]. However, *U. maydis* ITA Max has an additional mttA transporter (antiporter for malate and *cis*-aconitate in the mitochondrial membrane), while *U. maydis* Chassis has deletions in the mannosylerythritol lipid (MEL), ustilagic acid (UA) and diacylglycerol acyltransferase (dgat) enzymes. While all these deletions increase production, by either increasing the flux of pre-cursor molecules towards ITA (mttA) or directing more carbon towards the product (∆MEL ∆UA ∆dgat), both strains show similar production profiles. *U. maydis* Mutterschiff consolidates the genetic modifications of the other two strains into one, leading to further increased ITA production.

The difference between the two *U. cynodontis* strains can also be explained by the genetic modifications. The only metabolic difference between them is the mttA antiporter for malate and *cis-*aconitate, that is integrated into *U. cynodontis* ITA Max pH, allowing for increased ITA production.

The difference between *U. maydis* and *U. cynodontis* can, at least in part, be explained by the extended metabolic engineering of *U. maydis* Mutterschiff, compared to *U. cynodontis* ITA Max pH. Due to the deletion of by-product formation, higher ITA concentrations are expected for *U. maydis* Mutterschiff. As ITA production from sugars results in a net surplus of energy equivalents (NADH and ATP) [57], the difference in ITA titer between *U. maydis* and *U. cynodontis* cannot be due to a difference in the diversion of energy for growth and production alone. Therefore, the available carbon needs to be the limiting factor. As described before, a nitrogen limitation is necessary for ITA production [48]. Because both strains show product formation, all of the added nitrogen source must be consumed. Therefore, with the same amount of available nitrogen, the higher biomass for *U. cynodontis* can only be reached with a higher biomass yield per nitrogen, compared to *U. maydis*. Hence, less carbon is available for ITA production, because more is used for biomass, leading to lower ITA titers and yields. However, this drawback of *U. cynodontis* can be handled by accordingly adjusting the nitrogen content of the media, thus, allowing maximal ITA production and yield [39, 40].

In general, strains with higher specific production rates are considered good performers as they utilize less carbon for cell maintenance, due to shorter production times. Assuming the same constant maintenance coefficient for both, *U. maydis* Mutterschiff and *U. cynodontis* ITA Max pH, the substrate consumption for maintenance during the production phase is about 7.7 times higher with *U. cynodontis*, under the tested conditions (comp. Figure 1, Eq. 7). However, the assumptions of a constant maintenance coefficient that is the same for both strains under changing cultivation conditions might not hold true. This is already visible in the STR cultivations, where the difference in substrate consumption due to maintenance is estimated to be about 1.4-fold for *U. cynodontis* compared to *U. maydis* (comp. Figure 8 and S5, Eq. 7).

Both *U. cynodontis* strains have a lower OTR with higher OD than the *U. maydis* strains, showing a lower oxygen consumption per biomass. This is especially favourable in large-scale fermentations, where oxygenation is difficult and gradients arise [58].

Finally, *U. cynodontis* is more tolerant to acidic pH values, compared to *U. maydis* [36, 39], which might be interesting for the following unit operations in downstream processing. Nowadays, crystallisation at low pH is most commonly used [17, 59]. Therefore, using a strain that is able to withstand low pH values during the fermentation and, thus, reducing costs of pH adjusting agent and saline wastes, is beneficial [44]. Due to these reasons, *U. cynodontis* ITA Max pH was chosen for further process development.

The selection of a suitable feedstock is the next step of the process development. Growth and ITA production is possible on almost all tested crude feedstocks. However, the OTR increase indicates the availability of additional nitrogen in the complex substrates, which will negatively influence production. If the C/N ratio decreases (i.e. nitrogen increases), more carbon is used for biomass, up to a point, where no nitrogen limitation is reached, and no ITA is produced. Based solely on the ITA yield, the filtration retentate should be used for further analysis. However, the experiment does not show the full potential of the substrates due to the different C/N ratios. The observed higher titer with filtration retentate is due to the fact that almost three times as much sugar was available in the medium (see Table S1). This increases the C/N ratio, which is strongly influenced by the initially added NH_4_Cl, and leads to the increased ITA titer. Next to titer and yield, other aspects like tonnage, costs, and handling of the substrate need to be considered for the production process (Table S1). Due to the filtration retentate’s low availability and high costs (personal discussion with GNT Europa GmbH, Aachen, Germany), it was discarded as a possible substrate. The fruit preparation only showed very low ITA titers with *U. cynodontis* ITA Max pH, in addition to a high solid load and was, therefore, ruled out as well. Thin juice showed small solid loads and a comparable yield to thick juice, leaving the latter and molasses as the preferable carbon sources. HPLC analysis revealed, that both are mainly composed of sucrose and small amounts of the monomers glucose and fructose (data not shown). Molasses also contains significant amounts of lactate, likely due to acid fermentation during unsterile storage [47].

Growth of the production strain on the main carbon sources was then further analysed (Fig. 3). The OTR clearly shows the consumption of the three carbon sources glucose, fructose and lactate, with a preference for the sugar monomers glucose and fructose, consuming these before the acid. The change in pH further supports the assumption, that lactate is consumed by the microorganism. Additionally, lactate consumption has been previously described for *U. maydis* [60]. The experiment shows that *U. cynodontis* ITA Max pH can also metabolise lactate, which is necessary for an economical production process on molasses.

### Determination of biologically available nitrogen in molasses and thick juice

Nitrogen availability and the resulting C/N ratio play a significant role in the ITA production with Ustilaginaceae, due to the necessary nitrogen limitation [48]. Low nitrogen amounts have been shown to increase the yield, while simultaneously decreasing productivity [39, 40]. To optimise the production process, the available nitrogen amount in the complex substrate needs to be determined. Due to differences in metabolism between strains and the wide range of possible nitrogen sources, the biologically available nitrogen might differ from the total amount of nitrogen in the media. Therefore, the production strain was used for the determination. Due to the nitrogen limitation reached at OTR_N,max_, these values can be correlated with the ammonium concentration (Fig. 4). Using this correlation, the nitrogen content in the alternative feedstock can be estimated (Fig. 5). In addition, the OTR of the molasses cultivations, indicate the presence of a secondary nitrogen source (Fig. 5C). Because the strains behave the same with different ammonium concentrations until 18 h, this secondary nitrogen source must be consumed before the ammonium. The growth on the latter is, therefore, visible in the second increase of the OTR.

Even though the concentration of biologically available nitrogen is only twice as high in the molasses, compared to the thick juice, the amount of added nitrogen is much higher in a real production process. This is due to the lower sugar concentration and, therefore, C/N ratio in the molasses, leading to a higher volumetric ratio that needs to be added to the fermentation, in order to reach the desired sugar concentration. As higher nitrogen amounts are added with the feedstock molasses, thick juice was chosen as the substrate for the scale-up.

### Influence of osmolality, pH and ITA concentrations on *U. cynodontis* cultivations

Replacing pure sugars with complex substrates inevitably increases the osmolality of the medium, which can significantly influence the process [49]. Even though there is no difference in the OTR and biomass formation between the reference and osmolalities up to 850 mOsmol/kg, there is a difference in ITA titer and, therefore, yield. This can be explained by increased energy demand for maintenance. In addition, the biomass yield per nitrogen is reduced with higher osmolalities, as is evidenced by the decreasing OD. The increase in osmolality also corresponds to a lower growth rate in *U. cynodontis* ITA Max pH, as visible in the decreased slope of the OTR. However, the same effects are only in part visible for *U. maydis* Mutterschiff (Figure S3). While the growth rate shows a stronger dependency on the osmolality for this strain, the biomass and product yield remain constant with increasing osmolality. This is surprising, because previous data showed that *U. maydis* is only impacted to a small extend by osmolalities up to 2.5 osmol/L regarding growth [26]. However, Klement et al. worked with wildtype strains, while in this study highly engineered strains are used, which might have a lower robustness towards high osmolalities. A pH effect due to the higher buffer concentration with *U. maydis* Mutterschiff might also explain the difference between the two strains. To investigate this effect further, in addition to potential product inhibition, experiments with different initial ITA concentrations were conducted (Fig. 7). Here, a shift the in OTR peak with increasing initially added ITA concentration was observed. This is coherent with what was observed for increased osmolalities alone (Fig. 6). In addition, a decrease in the biomass with increasing initially added ITA, has also been seen for increased osmolality. However, the substantial decrease visible in Fig. 6B cannot be explained by an osmolality effect alone. Thus, the ITA itself negatively affects biomass formation, as has previously been described for *U. vetiveriae* in pH controlled stirred tank reactors [61]. Because additional ITA is formed under all tested conditions – indicating that a nitrogen limitation is reached – the biomass yield per available nitrogen is reduced, resulting in lower biomass formation. Therefore, more glucose is available for product formation, as seen in the increased ITA titers.

Next to osmolality, the pH is also critical for the production of weak acids. It has been shown that ITA concentrations are limited to ~ 80 g/L at low pH values by an effect called weak acid uncoupling [26, 39]. Compared to the reference, the higher pH with initially added ITA allows more ITA to be produced, because the acid is not fully protonated (pK_a_ 5.55 and 3.83) and, therefore, exhibits lower stress to the cells (Fig. 7) [47]. The reason for the higher pH presumably also lies in the initial ITA addition, which buffers the media at its pK_a_ value of 5.55.

The combination of reduced biomass and higher pH leads to increased ITA titers and, thus, increased yields. However, the calculated yield for the initial addition of 80 g/L ITA reaches 0.89 ± 0.01 g_ITA_/g_glucose_, which is considerably higher than the theoretical maximum of 0.72 g_ITA_/g_glucose_. This can be explained by evaporation in the flasks, leading to higher concentrations and, thus, to the observed high yields. This concentration effect is more substantial the higher the initial ITA concentration. However, due to the use of humidified air during the experiment, only a minimal volume change is to be expected. Thus, the observed trend of increasing yield with higher initial ITA concentrations can be attributed to higher pH values during the experiment. This is contrary to what has been shown for *U. maydis* wildtype [26]. There, lower amounts of ITA were produced with increased initial ITA concentration. However, CaCO_3_ was used as buffer, likely precipitating most of the initially added ITA [61]. Thus, the buffer capacity of the system was reduced dramatically, resulting in lower pH and stronger weak acid stress.

Based on these findings the biggest influence on itaconic acid production is the pH, due to weak acid stress, followed by osmolality and the itaconic acid itself. Therefore, a constant pH of 6.5 and an extended batch mode was used for the STR fermentation with *U. cynodontis* and thick juice as the sole carbon source.

### Extended batch stirred tank reactor cultivation of *U. cynodontis *ITA Max pH with thick juice as carbon source

Extended-batch cultivations have the same advantages of reducing the osmolality and increasing the production time as fed-batch cultivations, while allowing for maximum production rates [62]. Therefore, extended-batch mode was chosen.

In the beginning of the cultivation the high RQ indicates the production of a reduced product in addition to growth. These are likely intracellular lipids, which have previously been reported to be produced by members of the Ustilaginaceae family [63]. This is supported by the CDW, which increases during this time. At the same time, fructose starts to accumulate, likely due to catabolite repression of fructose uptake by glucose. Therefore, fructose accumulates while glucose is consumed. Once the OTR and CTR drop nitrogen limitation is reached (Fig. 8A) [39]. However, the CDW still continues to increase, which is likely due to a change in the biomass composition, which has previously been observed in *U. maydis* [26, 64]. Through the reduction of necessary nitrogen per carbon, more biomass is formed. This hypothesis is underlined by the RQ, which decreases to 0.9 between 28 h and the beginning of the feed phase, showing the metabolic shift from lipid formation and growth to ITA formation. Once the feed starts, nitrogen and carbon are fed into the reactor. Even though the CDW continues to increase, the ITA concentration also increases, showing that nitrogen is still limiting. The RQ of ~ 0.8 emphasises the hypothesis that nitrogen is limiting, thus, allowing ITA to be formed, even though growth is visible. After the feed ends, the RQ drops further showing stronger ITA formation with less growth compared to the feed phase. This is again supported by the increasing biomass, which is masked in the constant CDW by the dilution effect.

Carbon balance of the process showed that almost 20% of the carbon present in the products (ITA, biomass and CO_2_) is unaccounted for and does not originate from the sugars. This is not surprising, as thick juice likely contains further carbon sources like amino acids or other organic acids [44].

The reached yields are in the same range as the highest values previously published [39, 40]. Compared to reference fermentations on glucose at a constant pH of 3.6, the titer and yield increased by 139% and 28%, respectively, for *U. cynodontis* ITA Max pH [39]. The increase of both ITA titer and yield is likely due to the lower product toxicity at higher pH. The reference cultivation was performed at pH 3.6, which leads to a higher ratio of protonated to unprotonated ITA and, thus, stronger weak acid stress [22]. The pH of 6.5, therefore, allows ITA titers above ~ 80 g/L. However, for an optimised process, the purification method of ITA has to be taken into account. Recently, it has been shown that lower titers due to low pH are more cost-effective, if the next unit operation demands a low pH, like crystallisation [44]. A low pH during the fermentation drastically reduced the costs for pH adjusting agents and saline waste, improving the economy of the process. Furthermore, inherent challenges to the process, like osmolality and the presence of ITA, need to be addressed in future research. *In-situ* product removal shows great potential, as it reduces the ITA concentration and the osmolality, allowing for an increased production [59]. Alternatively, adaptive laboratory evolution experiments have increased the acid tolerance of *Saccharomyces cerevisiae* and could be used to induce the same effect for the Ustilaginaceae strains [65]. In addition, the fermentation medium could be further optimized by reducing the initially added components, as some of them might be contained in large excess and are added through the alternative feedstock. Lastly, the production process as a whole can be further optimised, for example regarding optimal pH and purification route.

## Conclusion

In this study, *U. maydis* and *U. cynodontis* strains were used to produce ITA on complex substrates from the local food industry. A suitable substrate was identified using the newly developed µTOM device for microtiter plates. Here, nitrogen availability and C/N ratios were of key importance. A method was developed to determine the biologically available nitrogen, which can be used for any other component of a (complex) substrate. This method enables rapid screening of the suitability of different kinds or batches of substrates for production.

The effects of complex substrate characteristics like osmolality, pH, and product inhibition, were examined in shake flasks. Based on these, a successful scale-up to 2 L fermenter was performed with thick juice as the sole carbon source at a constant, high pH. This study demonstrates the use of alternative, complex feedstocks and waste products from the local food industry for the production of ITA with Ustilaginaceae, without drawbacks in either titer or yield, compared to glucose fermentations.

## Materials and methods

### Microbial strains and media composition

*Ustilago cynodontis* NBRC9727 ∆fuz7r ∆cyp3r P_ria1_ria1 (*U. cynodontis* pre-gen), *Ustilago cynodontis* NBRC9727 ∆fuz7r ∆cyp3r P_etef_mttA P_ria1_ria1 (*U. cynodontis* ITA Max pH) [36], *Ustilago maydis* MB215 ∆cyp3 ∆P_ria1_::P_etef_ ∆fuz7 P_etef_mttA (*U. maydis* ITA Max) [37], *Ustilago maydis* MB215 2229 ∆cyp3 ∆MEL ∆UA ∆dgat ria1↑ ∆fuz7 (*U. maydis* ITA chassis) [38] and *Ustilago maydis* MB215 2229 ∆cyp3 ∆MEL ∆UA ∆dgat ria1↑ ∆fuz7 mttA↑ (*U. maydis* Mutterschiff) [40] were used for the cultivation experiments. The strains were stored as cryogenic cultures at -80 °C, containing 30% (v/v) of 500 g/L glycerol stock and 70% (v/v) culture grown on modified Verduyn medium (see below), containing 50 g/L glucose and 4 g/L NH_4_Cl. Cultivations were performed in modified Verduyn medium as described before by Geiser et al. [32]. If not stated otherwise, the medium was composed of 0.2 g/L MgSO_4_·7 H_2_O, 0.01 g/L FeSO_4_·7 H_2_O, 0.5 g/L KH_2_PO_4_ and 0.1% (v/v) trace element solution. The trace element solution consisted of 15 g/L TitriplexIII^©^, 4.5 g/L ZnSO_4_·7 H_2_O, 0.84 g/L MnCl_2_·2 H_2_O, 0.3 g/L CoCl_2_·6 H_2_O, 0.3 g/L CuSO_4_·5 H_2_O, 0.4 g/L Na_2_MoO_4_·2 H_2_O, 4.5 g/L CaCl_2_·2 H_2_O, 3 g/L FeSO_4_ · 7 H_2_O, 1 g/L H_3_BO_3_ and 0.1 g/L KI. Flask and MTP experiments additionally contained 0.1 or 0.03 M 2-(N-morpholino) ethane sulfonic acid (MES), while 1 g/L yeast extract and 0.1% (v/v) vitamin solution were added to stirred tank reactor cultivations. The vitamin solution consisted of 0.05 g/L D-biotin, 1 g/L D-calcium pantothenate, 1 g/L nicotic acid, 25 g/L myoinositol, 1 g/L thiamine hydrochloride, 1 g/L pyridoxal hydrochloride, and 0.2 g/L para-aminobenzoic acid. Added concentrations of carbon sources and NH_4_Cl are individually given for each experiment. The media components were either sterile-filtered with a 0.2 μm cut-off filter (Millipore-Sigma, Burlington, USA) or autoclaved at 121 °C for 20 min.

An overview for the feedstocks used for Fig. 2 and S2 is provided in Table S1.

The alternative feedstocks were kindly provided by Pfeifer & Langen Industrie- und Handel-KG (molasses, thin and thick juice), Zentis GmbH & Co. KG (fruit preparations) and GNT Europe GmbH (filtration retentate and permeate). Molasses, thin and thick juice come from the sugar manufacturing process as side (thin and thick juice) or waste streams (molasses). The “fruit preparation” is a waste stream coming from jam and marmalade production. Filtration retentate and permeate are both side streams in the pigment extraction from carrots.

### Cultivation in shaken systems

Cultivations on small scale were carried out either in 96 round deep-well MTP (riplate RW, 2.0 ml round deepwell plate, HJ-BIOANALYTIK GmbH, Erkelenz, Germany) or in 250 mL Respiratory Activity MOnitoring System (RAMOS) shake flasks at 350 rpm, with a shaking diameter of 50 mm at 30 °C (Climo-Shaker ISF1-X, Kuhner, Birsfelden, Switzerland). Filling volumes ranged from 300 µL in MTP to 10 mL in shake flasks. For sterility, MTP were covered with gas-permeable sealing films (AeraSeal™, Sigma-Aldrich, St. Louis, USA). Online monitoring of the oxygen transfer rates was achieved using the RAMOS for shake flasks and the newly developed µTOM for MTP [50, 54, 66].

Pre-cultures were inoculated with 1 mL cryogenic stock (OD 5) per 10 mL pre-culture medium and cultivated overnight. They were then centrifuged at 14.000 revolutions per minute (rpm) for 5 min at room temperature and resuspended in the main culture medium. Main cultures were inoculated to an OD of 0.8.

### Cultivations in stirred tank bioreactors

Cultivations were performed in 2 L Sartorius BIOSTAT^®^ stirred tank reactors (Sartorius AG, Göttingen, Germany) equipped with 4 baffles and one six-blade Rushton turbine. Fermentations were performed with an initial filling volume of 1 L modified Verduyn medium with 200 g/L sucrose from thick juice and 4 g/L NH_4_Cl at 30 °C. During the feed phase, additional 200 g of sucrose in the form of thick juice was fed into the reactor. The dissolved oxygen tension (DOT) was kept > 30% by increasing the stirring frequency from 800 to 1200 rpm, while pH was maintained at 6.5 by addition of 5 M NaOH and 1 M HCl. Oxygen and carbon dioxide transfer rates were determined by measuring the off-gas with a DASGIP^®^ off-gas analyser GA4 (Eppendorf SE, Hamburg, Germany). pH and DOT were measured using an Easyferm plus PHI K8 200 (Hamilton, Hoechst, Germany) and a VisiFerm DO ECS 225 probe (Hamilton, Hoechst, Germany), respectively. 0.05% (v/v) antifoam 204 (Sigma-Aldrich, St. Louis, USA) was added at the beginning of the fermentation, and when necessary, to prevent foaming.

One YEPS plate containing 10 g/L yeast extract, 10 g/L peptone, 10 g/L sucrose, and 20 g/L agar-agar was inoculated from a fresh cryogenic vial and cultivated for 48 h at 30 °C for each experiment. Three individual 500 mL shake flasks with 50 mL modified Verduyn medium containing 50 g/L sugar from thick juice and 2 g/L NH_4_Cl were inoculated as pre-culture from the plate and cultivated overnight at 30 °C, 300 rpm, and a shaking diameter of 50 mm. To reduce the inoculation volume, the filling volume of each flask was then centrifuged (4,000 rpm, 10 min). The pellets were each resuspended in 3 mL of the supernatant and combined. The fermenter was then inoculated to an OD of 0.25.

### Offline analytics

Samples were analysed regarding sucrose, fructose, glucose and itaconic acid concentration using high-performance liquid chromatography (HPLC). Samples were centrifuged at 15,000 rpm for 10 min, the supernatant was diluted appropriately with deionised water and then filtered with a 0.2 μm cut-off filter (Millipore-Sigma, Burlington, USA). HPLC analysis was performed in three dilutions per biological sample using a Thermo Fisher Ultimate 3000 (Thermo Fisher Scientific Inc., Waltham, USA) equipped with an ERC RefractoMax 520 RID (Shodex, Munich, Germany). Separation was achieved with a ROA-Organic Acid H+ (8%) (300 × 7.8 mm) column (Phenomenex, Torrance, USA) heated to 30 °C and a mobile phase of 5 mM H_2_SO_4_ running at 0.8 mL/min. OD was measured using a Genesys 20 photometer (Thermo Fisher Scientific Inc., Waltham, USA) at a wavelength of 600 nm. pH was measured with a HI221 Basic pH (Hanna Instruments Deutschland GmbH, Voehringen, Germany). Osmolality was determined with a cryoscopic Osmometer OSMOMAT^®^ 030 (Genotec, Berlin, Germany).

For the STR cultivations, the cell dry weight was determined by weighing dried cell pellets of three 2 mL samples (centrifuged at 14,000 rpm for 10 min, dried at 80 °C for 48 h, cooled for at least 30 min in a desiccator) in 2 mL Eppendorf tubes for each sample point. The tubes without cells were dried and weighed before samples were added (dried at 80 °C for 48 h, cooled for at least 30 min in a desiccator).

### Calculations

As key performance indicator, the yield [g_ITA_/g_sugar_] was calculated based on the produced ITA [g] and the consumed sugar mass [g] of sucrose, glucose and fructose. Other compounds in the complex feedstock were not considered. For the STR fermentation, the samples taken during the cultivation were considered for the mass balance. Equation 1 shows the calculation of the mass balances for the substrates.

1 $${m_x}\left( t \right) = {c_x}\left( {{t_0}} \right) \cdot {V_L}\left( {{t_0}} \right) + {c_{x,feed}} \cdot {V_{feed}}\left( t \right) - \sum\nolimits_{{t_0}}^t {{c_x}(t)} \cdot {V_{sample}}\left( t \right)$$

Here, ${m}_{x}\left(t\right)$ [g] is the calculated mass at time point t, ${c}_{x}\left({t}_{0}\right)$ [g/L] is the concentration of the different substrates (glucose, fructose or sucrose) at the beginning of the fermentation, ${V}_{L}\left({t}_{0}\right)$ [L] is the initial filling volume, ${c}_{x,feed}$ [g/L] is the concentration of the substrate in the feed and ${V}_{feed}\left(t\right)$ [L] is the added feed volume at time point t. To consider sampling, the sum of the substrate concentrations (${c}_{x}\left(t\right)$ [g/L]) times the sample volume (${V}_{sample}\left(t\right)$ [L]) is subtracted. To calculate the mass balance for the produced ITA Eq. 2 is used.

2 $${m_{ITA}}\left( t \right) = {c_{ITA}}\left( t \right) \cdot {V_L}\left( t \right) + \sum\nolimits_{{t_0}}^t {c{}_{ITA}(t)} \cdot {V_{sample}}\left( t \right)$$

The mass of ITA [g] is calculated by multiplying the ITA concentration ${c}_{ITA}\left(t\right)$ [g/L] by the filling volume ${V}_{L}\left(t\right)$ [L] at time point t. The sum of the ITA concentrations ${c}_{ITA}\left(t\right)$ [g/L] times the sample volume ${V}_{sample}\left(t\right)$ [L] considers the sampling.

The yield can then be calculated by dividing the mass of ITA [g] by the sum of the substrates glucose [g], fructose [g], and sucrose [g] (Eq. 3).

3 $$Y\left( t \right) = {m_{ITA}}\left( t \right) \cdot {\left( {{m_{glucose}}\left( t \right) + {m_{fructose}}\left( t \right) + {m_{sucrose}}\left( t \right)} \right)^{ - 1}}$$

Yields on glucose were calculated by counting one mol of sucrose as two mole of glucose.

For the STR fermentations, the space-time-yield (STY) was calculated by dividing the mass of produced ITA [g] by the sum of the filling volume of the fermenter [L] and the total sample volume [L] times time [h] (Eq. 4).

4 $$STY\left( t \right) = {m_{ITA}}\left( t \right) \cdot {({V_L}\left( t \right) + {V_{sample,total}}\left( t \right))^{ - 1}} \cdot {t^{ - 1}}$$

The respiratory quotient [-] is a crucial fermentation parameter, which allows drawing conclusions on the microorganisms’ behaviour. The RQ is calculated by dividing the CTR by the OTR, according to Eq. 5.

5 $$RQ = CTR \cdot OT{R^{ - 1}}$$

For all the above mentioned calculations errors are based on gaussian error propagation, when applicable.

Statistical analysis tools (*scypi.stats* and *statsmodels* modules in *Python 3.0*) were used to determine significance of the data from experiments with biological triplicates (Figs. 1 and 2, OD, ITA titer, and yield). Normal distribution was assumed and homogeneity of variance was determined using Levene’s test. Afterwards, an ANOVA with a significance level of α = 0.05 was used to determine, whether there is a significant difference between the different strains. As all tested conditions showed a significant difference, Tukey’s Honestly Significant Difference test was performed, to determine between which groups the difference was significant. As statistical significance was determined between almost all groups, only the ones, where no difference was determined, are mentioned in the manuscript.

When comparing fermentation with different durations, the maintenance has to be considered. The longer cultivation requires more carbon for maintenance, thus, lowering the substrate yield. The amount of substrate used for maintenance during the production phase for *U. cynodontis* ITA Max pH and *U. maydis* Mutterschiff was estimated for experiments depicted in Figs. 1 and 8 and S5. In the following calculation, only that part of the fermentations, which are nitrogen limited and where there is no substantial growth, is considered. Assuming a constant biomass (${m}_{bio}$) for the whole production phase, the consumed substrate due to maintenance (${c_{S,m}}$) [g] can be calculated according to Eq. 6.

6 $${c_{S,m}} = {m_{bio}} \cdot {m_S} \cdot {t_{prod}}$$

With ${m}_{bio}$ as biomass [g], ${m}_{S}$ as maintenance coefficient [g_substrate_/g_biomass_/h] and ${t}_{prod}$ as production time [h]. Further, assuming the same, constant maintenance coefficient for both strains, the ratio of ${c_{S,m}}{(r_{m})}$ [-] between the strains can be calculated according to Eq. 7.

7 $${r_m} = {m_{bio,U.cynodontis}} \cdot {t_{prod,U.cynodontis}} \cdot {\left( {{m_{bio,U.maydis}} \cdot {t_{prod,U.maydis}}} \right)^{ - 1}}$$

With ${m}_{bio,U. cynodontis}$ as biomass for *U. cynodontis* ITA Max pH [g or -], ${t}_{prod, U. cynodontis }$ as the production time of *U. cynodontis* ITA Max pH [h], ${m}_{bio,U. maydis}$ as biomass for *U. maydis* Mutterschiff [g or -] and ${t}_{prod, U. maydis }$ as the production time of *U. maydis* Mutterschiff [h]. Note that no specific values for ${c_{S,m}}$ are calculated and, therefore, OD can be used for ${m}_{bio}$. In addition, no maintenance coefficient is required.

The carbon balance includes the sugars sucrose, fructose and glucose, in addition to the products itaconic acid, biomass and CO_2_. For all included compounds, except CO2, the balance is based on the mass calculations above (Eqs. 1 and 2). C-mol was calculated by dividing the mass by the molar mass and multiplying by the number of carbons of the respective molecule. CO_2_ was directly measured in C-mol with the off-gas analyser (see “Cultivations in stirred tank reactors”).The biomass composition was based on Klement et al. [26].

### Electronic supplementary material

Below is the link to the electronic supplementary material.

Supplementary Material 1

## Data Availability

The datasets supporting the conclusions of this article are included within the paper and the additional file (Figures S1 – S5, Table S1 - S2). The datasets used and analysed during the study are available from the corresponding author upon reasonable request.

## Abbreviations

CDW: Cell dry weight
CTR: Carbon dioxide transfer rate
*dgat*: Diacylglycerol acyltransferase
DOT: Dissolved oxygen tension
HPLC: High performance liquid chromatography
ITA: Itaconic acid
KPI: Key performance indicator
MEL: Mannosylerythritol lipid
MES: 2-(N-morpholino) ethane sulfonic acid
MTP: Microtiter plate
OD: Optical density at 600 nm
OTR: Oxygen transfer rate
OTR_N,max_: Maximal obtained oxygen transfer rate
RAMOS: Respiratory Activity MOnitoring System
rpm: Revolutions per minute
RQ: Respiratory quotient
STR: Stirred tank reactor
UA: Ustilagic acid
