# Supplementary material for: Development of an itaconic acid production process with Ustilaginaceae on alternative feedstocks
Source: BMC Biotechnol. 2023 Sep 3;23:34. doi: 10.1186/s12896-023-00802-9 (PMC10476437; doi:10.1186/s12896-023-00802-9)
Supplement: Supplementary file 1 — Supplementary Material 1 [file 12896_2023_802_MOESM1_ESM.docx]

**Supplementary Figures**


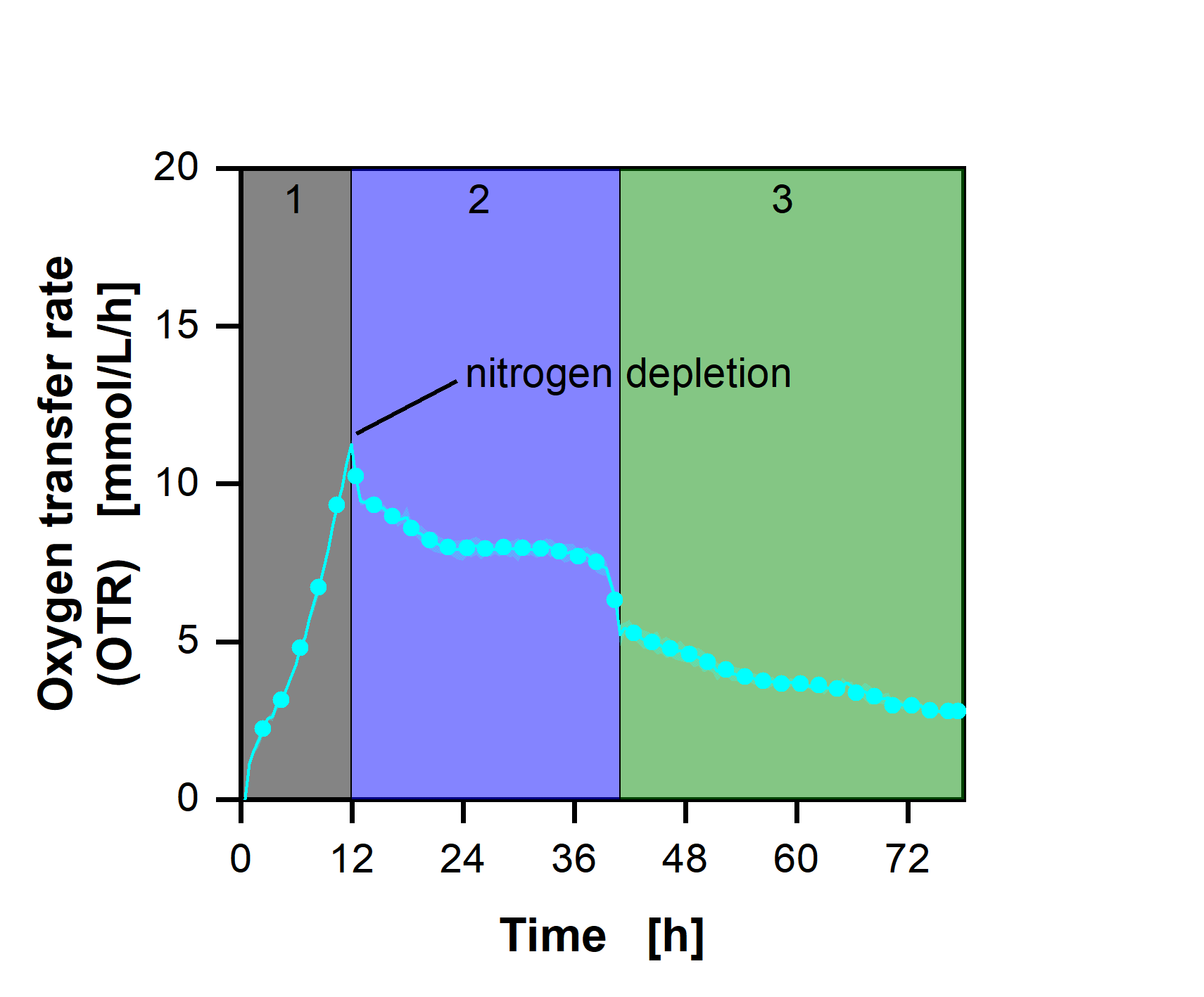


**Figure S1: Typical phases of itaconic acid production by Ustilaginacea grown on glucose with limiting ammonium chloride concentration (1 g/L).** Due to the similarity of these phases, only U. cynodontis ITA Max pH is shown as an example. Cultivation phases are indicated by number. 1: Exponential growth phase. 2: Nitrogen limited production phase. 3: Carbon depletion and starvation. Cultivations were performed in a 96 round deep-well MTP, filled with 300 µL modified Verduyn medium with 25 g/L glucose at 30 °C, 350 rpm shaking frequency and a shaking diameter of 50 mm. 30 mM MES were added to the cultivations. The initial pH was set to 6.5 for all cultivations. The graph shows the mean of three replicates, with standard deviation as shaded area. Due to high reproducibility of the measurements, the standard deviation might not be visible for every data point. For clarity, only every fourth data point is shown.

**
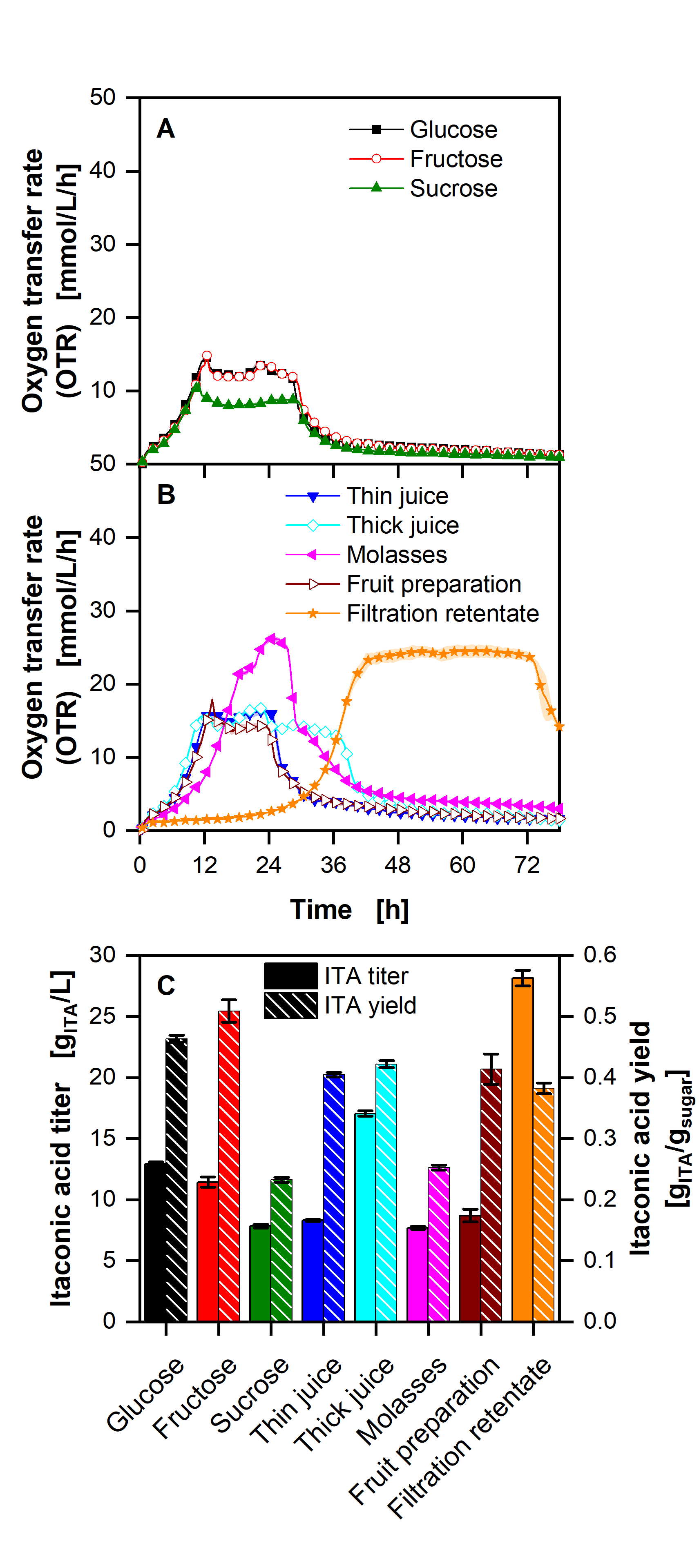
**

**Figure S2: Cultivation of U. maydis Mutterschiff with different carbon sources with limiting ammonium chloride concentrations (1 g/L).** (A) Oxygen transfer rates of the pure sugars (glucose, fructose and sucrose). (B) Oxygen transfer rates of complex substrates. (C) Itaconic acid titers and yields of the cultivations after 78 hours of cultivation. Cultivations were performed in a 96 round deep-well MTP, filled with 300 µL modified Verduyn medium at 30 °C, 350 rpm shaking frequency and a shaking diameter of 50 mm. 100 mM MES were added to the cultivations. The initial pH was set to 6.5 for all cultivations. The different carbon sources are specified in Table 1. Graphs show the mean of three replicates, with standard deviation as shaded area. Due to high reproducibility of the measurements, the standard deviation might not be visible for every data point. For clarity, only every fourth data point is shown.


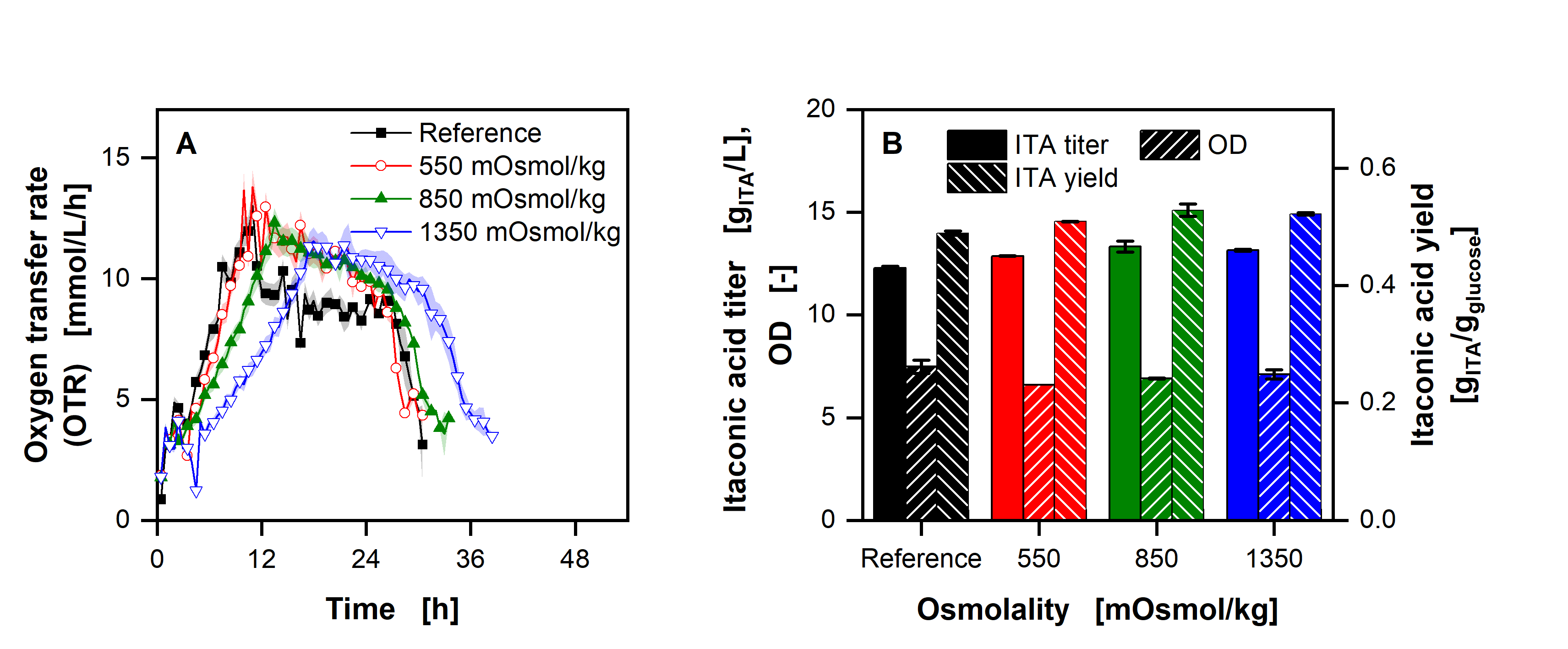


**Figure S3: Influence of osmolality on growth and productivity of U. maydis Mutterschiff with limiting ammonium chloride concentration (1 g/L NH_4_Cl).** (A) Course of the oxygen transfer rates with increasing osmolality. (B) Itaconic acid titer, OD and yield at the end of the respective fermentations. Osmolality was increased by addition of 0.1, 0.25 and 0.5 M NaCl. The reference medium has an osmolality of 350 mOsmol/kg. Cultivations were performed in 250 mL RAMOS flasks filled with 10 mL modified Verduyn medium with 25 g/L glucose at 30 °C, 350 rpm shaking frequency and a shaking diameter of 50 mm. 100 mM MES were added to the cultivations. The initial pH was at 6.5 for all cultivations. The final pH reaches 3.71 ± 0.01, 3.57 ± 0.00, 3.57 ± 0.01 and 3.45 ± 0.01 for the reference, 550, 850 and 1350 mOsmol/kg cultivations, respectively. Graphs show the mean of duplicates. Shadows and error bars show the minimum and maximum values. For clarity, only every second data point is shown.


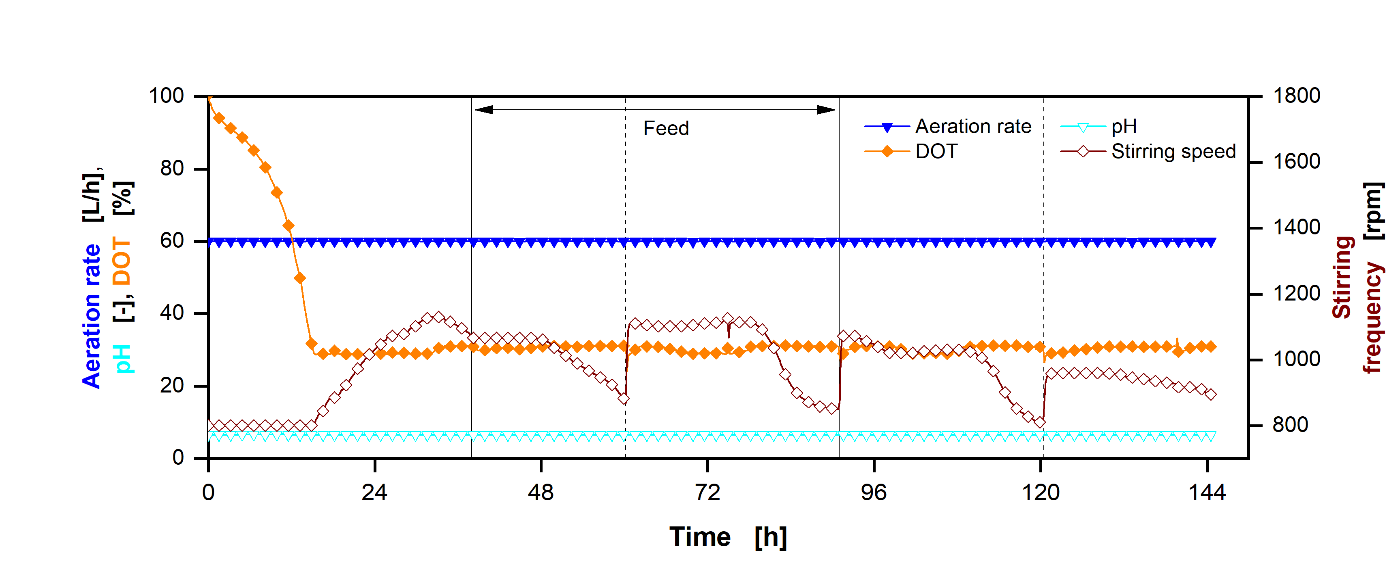


**Figure S4: Absolute aeration rate, pH, dissolved oxygen tension (DOT) and stirring frequency of the extended-batch fermentation of U. cynodontis ITA Max pH (Figure 8).** For the batch phase, 200 g/L sucrose from thick juice were initially added to the medium. During the feed phase (between the vertical solid lines) 200 g of additional sucrose in form of thick juice were added into the fermentation vessel. Vertical dotted lines show the addition of antifoam. In addition, antifoam was added at the end of the feed phase. Cultivation was performed in a 2 L Sartorius BIOSTAT® stirred tank reactor (Sartorius AG, Göttingen, Germany) with an initial filling volume of 1 L at 30 °C. Dissolved oxygen tension was kept > 30 % by increasing the stirring frequency from 800 to 1200 rpm. pH was kept constant at 6.5 by addition of 1 M HCl and 5 M NaOH. For clarity, only every tenth data point is shown.


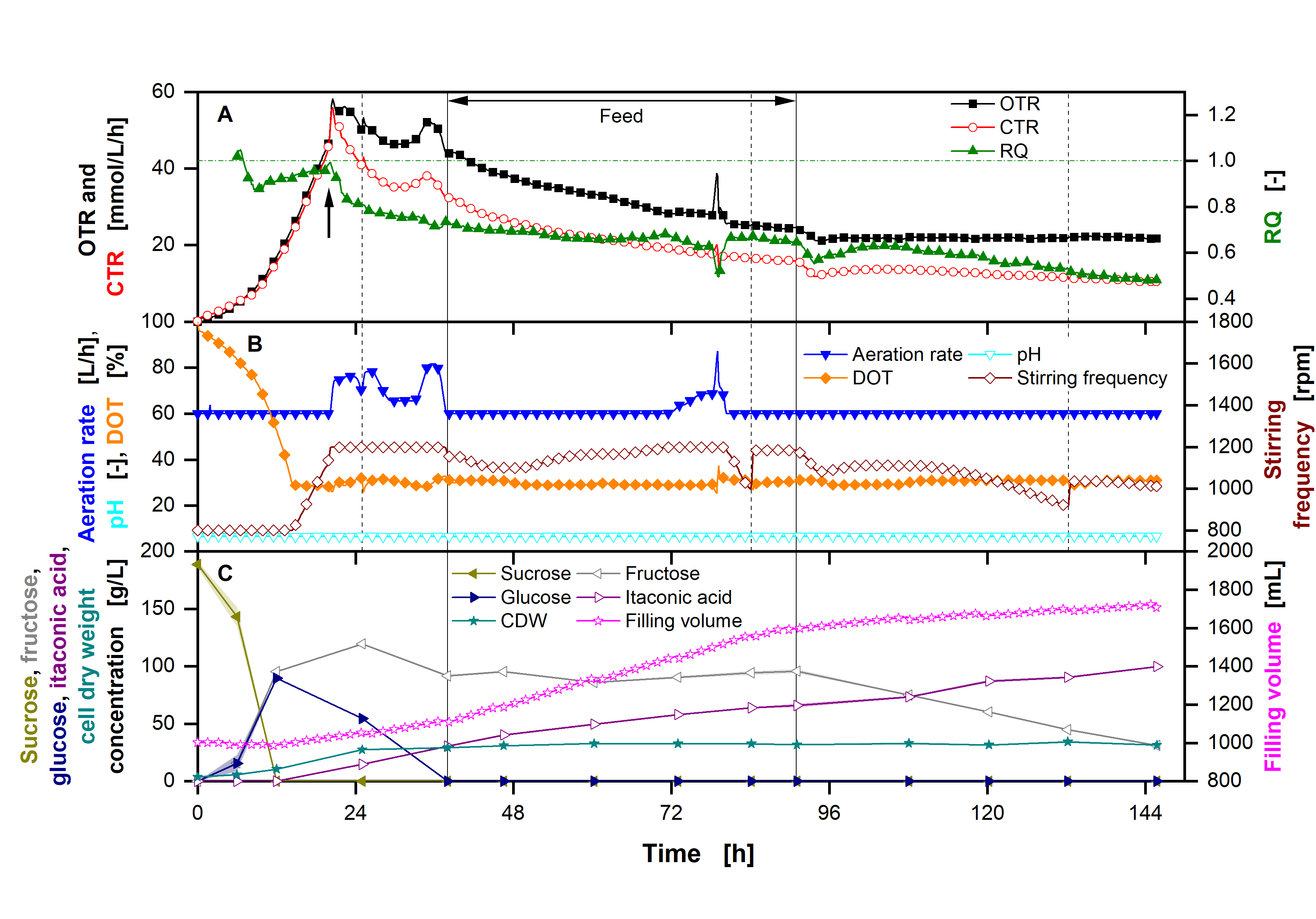


**Figure S5: Extended-batch fermentation of U. maydis Mutterschiff grown with thick juice as sole carbon source with limiting ammonium chloride concentration (4 g/L NH_4_Cl).** (A) Oxygen and carbon dioxide transfer rates (OTR, CTR) and respiratory quotient (RQ). The black arrow indicates the start of the nitrogen limitation. The green horizontal dotted-dashed line indicates an RQ = 1. (B) Absolute aeration rate, pH, dissolved oxygen tension (DOT) and stirring frequency. (C) Sugar (sucrose, glucose, fructose) and product concentrations (itaconic acid (ITA), cell dry weight (CDW)) and filling volume. For the batch phase, 200 g/L sucrose from thick juice were initially added to the medium. During the feed phase (between the vertical solid lines) 200 g of additional sucrose in form of thick juice were added into the fermentation vessel. Vertical dotted lines show the addition of antifoam. Cultivation was performed in a 2 L Sartorius BIOSTAT® stirred tank reactor (Sartorius AG, Göttingen, Germany) with an initial filling volume of 1 L at 30 °C. Dissolved oxygen tension was kept > 30 % by first increasing stirring frequency from 800 to 1200 rpm and second the gas flow rate from 60 to 180 L/h. pH was kept constant at 6.5 by addition of 1 M HCl and 5 M NaOH. RQ values are only shown for OTR values > 5 mmol/L/h. Samples were taken regularly and analyzed via HPLC. Concentrations are shown as mean values of three replicates and standard deviation as shaded area. Due to high reproducibility of the measurements, the standard deviation might not be visible for every data point. For clarity, only every tenth data point is shown for the online data.

**Supplementary Tables**

**Table S1: Feedstocks used for Figure 2 and S2**

| **Feedstock** | **Sugar concentration [g/L]** | **Availability** | **Handling** | **Cost** | **Manufacturer** |
| --- | --- | --- | --- | --- | --- |
| Glucose | 27.9 | High | Easy | High | Sigma-Aldrich |
| Fructose | 22.5 | High | Easy | High | Sigma-Aldrich |
| Sucrose | 33.7 | High | Easy | High | Sigma-Aldrich |
| Thin juice | 20.5 | High | Difficult (solids) | Low | Pfeifer & Langen Industrie- und Handel-KG |
| Thick juice | 40.4 | High | Easy | Low | Pfeifer & Langen Industrie- und Handel-KG |
| Molasses | 32.0 | High | Difficult (viscosity) | Low | Pfeifer & Langen Industrie- und Handel-KG |
| Fruit preparation | 21.0 | High | Difficult (solids) | Low | Zentis GmbH & Co. KG |
| Filtration retentate | 93.6 | Low | Easy | High | GNT Europe GmbH |
| Filtration permeate | 65.8 | Low | Easy | High | GNT Europe GmbH |

**Table S2: Carbon balance of the stirred tank reactor fermentation with U. cynodontis ITA Max pH on thick juice as sole carbon source.**

|  |  | **C-mol [mol]** | **Balance [%]** |
| --- | --- | --- | --- |
| Substrates | Initial sugars | 7.41 | 51.4 |
|  | Feed sugars | 7.00 | 48.6 |
| Products | Itaconic acid | 7.70 | 53.4 |
|  | Biomass | 4.86 | 33.8 |
|  | CO_2_ | 4.67 | 32.4 |
|  | Unaccounted for | -2.82 | -19.6 |
